# Emerging encapsulation techniques for controlled and targeted delivery of bioactive compounds in food and nutraceutical systems

**DOI:** 10.3389/fnut.2026.1826702

**Published:** 2026-05-26

**Authors:** Komal Kumari, Soubhagya Tripathy, Prem Prakash Srivastav

**Affiliations:** Department of Agricultural and Food Engineering, Indian Institute of Technology Kharagpur, Kharagpur, West Bengal, India

**Keywords:** advanced encapsulation, bioactive compounds, complex coacervation, controlled release, liposomes, starch

## Abstract

Bioactive compounds have attracted considerable attention for their health-promoting properties, including antioxidant, anti-inflammatory, antimicrobial, and disease-preventive effects. However, their practical application in food and nutraceutical systems is often limited by poor physicochemical stability, low solubility, sensitivity to environmental stress, and low bioavailability during gastrointestinal digestion. Encapsulation technologies have emerged as a promising strategy to overcome these limitations by protecting bioactive compounds from degradation, enhancing their stability, and enabling controlled and targeted release. This review provides a comprehensive overview of advanced encapsulation materials and techniques used for improving the delivery efficiency of bioactive compounds. Various encapsulating materials, including polysaccharides, proteins, and lipid-based systems, are discussed in terms of their structural properties, encapsulation mechanisms, and functional advantages. The article further highlights emerging encapsulation technologies, such as ionic gelation, electrospinning, complex coacervation, and liposome-assisted delivery systems, which have shown significant potential to improve the encapsulation efficiency, stability, and bioaccessibility of sensitive bioactive compounds. In addition, recent advances in targeted and stimuli-responsive delivery systems are explored, emphasizing their ability to release bioactive compounds under specific physiological conditions. Advanced encapsulation strategies provide effective solutions to enhance the stability, bioavailability, and functional performance of bioactive compounds, supporting the development of functional foods and nutraceutical products.

## 1 Introduction

Bioactive compounds have attracted significant attention in recent years due to their diverse health-promoting properties, including antioxidant, anti-inflammatory, antimicrobial, and anticancer activities (1, 2). These compounds play an essential role in the development of functional foods, nutraceuticals, and pharmaceutical formulations aimed at improving human health and preventing chronic diseases (3). Growing consumer awareness regarding the relationship between diet and health has accelerated the demand for functional ingredients enriched with natural bioactive compounds in food and nutraceutical products (4). Consequently, extensive research efforts have focused on improving the stability, delivery efficiency, and biological activity of these compounds in complex food systems (5). Despite their considerable health benefits, many bioactive compounds exhibit poor physicochemical stability and low bioavailability, which significantly restrict their practical application in food and nutraceutical products (6). Many phytochemicals are highly sensitive to environmental factors, including oxygen, light, moisture, temperature fluctuations, and pH changes, which may lead to rapid degradation and loss of biological activity during processing and storage (7). Furthermore, several bioactive compounds exhibit poor water solubility and limited gastrointestinal stability, leading to reduced absorption and bioaccessibility in the human body (8). These limitations present significant challenges for the incorporation of bioactive compounds into functional food matrices and nutraceutical formulations (9).

Encapsulation technology has emerged as one of the most effective strategies to overcome these challenges by entrapping bioactive compounds within protective carrier materials (1, 10). Encapsulation not only protects sensitive compounds from environmental degradation but also improves their stability, solubility, and controlled release in targeted physiological environments (11). By protecting bioactive compounds from adverse external conditions, encapsulation systems can significantly enhance their shelf life, functional performance, and bioavailability in food and nutraceutical applications (12, 13). In addition, encapsulation technologies enable the controlled and targeted delivery of bioactive molecules within the gastrointestinal tract, thereby improving their absorption and therapeutic efficacy (6, 14). Various encapsulating materials have been explored for the design of efficient delivery systems, including natural polysaccharides, proteins, lipids, and composite biopolymers (5). Among these, polysaccharides such as starch, pectin, cellulose, alginate, and chitosan are widely used due to their biocompatibility, biodegradability, and ability to form stable matrices that can entrap bioactive molecules (15, 16). Protein-based carriers such as whey proteins, caseins, gelatin, and soy proteins are also commonly employed due to their excellent emulsifying, gelling, and film-forming properties, which facilitate the development of stable encapsulation systems (17, 18). In addition, lipid-based carriers such as liposomes, nanoemulsions, and structured lipid particles have demonstrated considerable potential for improving the delivery of hydrophobic bioactive compounds by enhancing their solubility and gastrointestinal stability (19).

Recent advances in food nanotechnology and biomaterials engineering have further expanded the scope of encapsulation systems by enabling the development of nano- and micro-scale delivery platforms with improved functional performance (20). These advanced delivery systems include nanoparticles, nanofibers, nanogels, nanoemulsions, and multilayered structures, which offer high encapsulation efficiency and controlled release (21). Among emerging approaches, techniques such as ionic gelation, electrospinning, complex coacervation, and liposome-assisted encapsulation have attracted considerable attention for their ability to produce stable, efficient delivery systems for sensitive bioactive compounds (22). These technologies allow precise control over particle size, morphology, and release kinetics, thereby enhancing the stability and bioaccessibility of encapsulated compounds (23). In addition to conventional encapsulation systems, stimuli-responsive and targeted delivery platforms have emerged as innovative strategies to enhance the functional performance of bioactive compounds (24). These “smart delivery systems” can respond to environmental triggers such as pH, temperature, enzymatic activity, or redox conditions, enabling site-specific release of encapsulated compounds within the gastrointestinal tract or targeted tissues (25). Such advanced systems not only enhance the bioavailability of bioactive compounds but also minimize premature degradation and improve their therapeutic potential (26).

Therefore, the objective of this review is to provide a comprehensive overview of advanced encapsulation materials and emerging technologies used to enhance the stability, bioavailability, and controlled delivery of bioactive compounds in food and nutraceutical systems. Furthermore, the review discusses advanced encapsulation techniques, including ionic gelation, electrospinning, complex coacervation, and liposome-assisted delivery systems, and emphasizes their roles in improving encapsulation efficiency and controlling release behavior. Additionally, recent progress in targeted and stimuli-responsive delivery systems is examined to provide insights into innovative strategies for developing next-generation delivery platforms for functional food and nutraceutical applications.

## 2 Encapsulating materials

**Figure 1:**
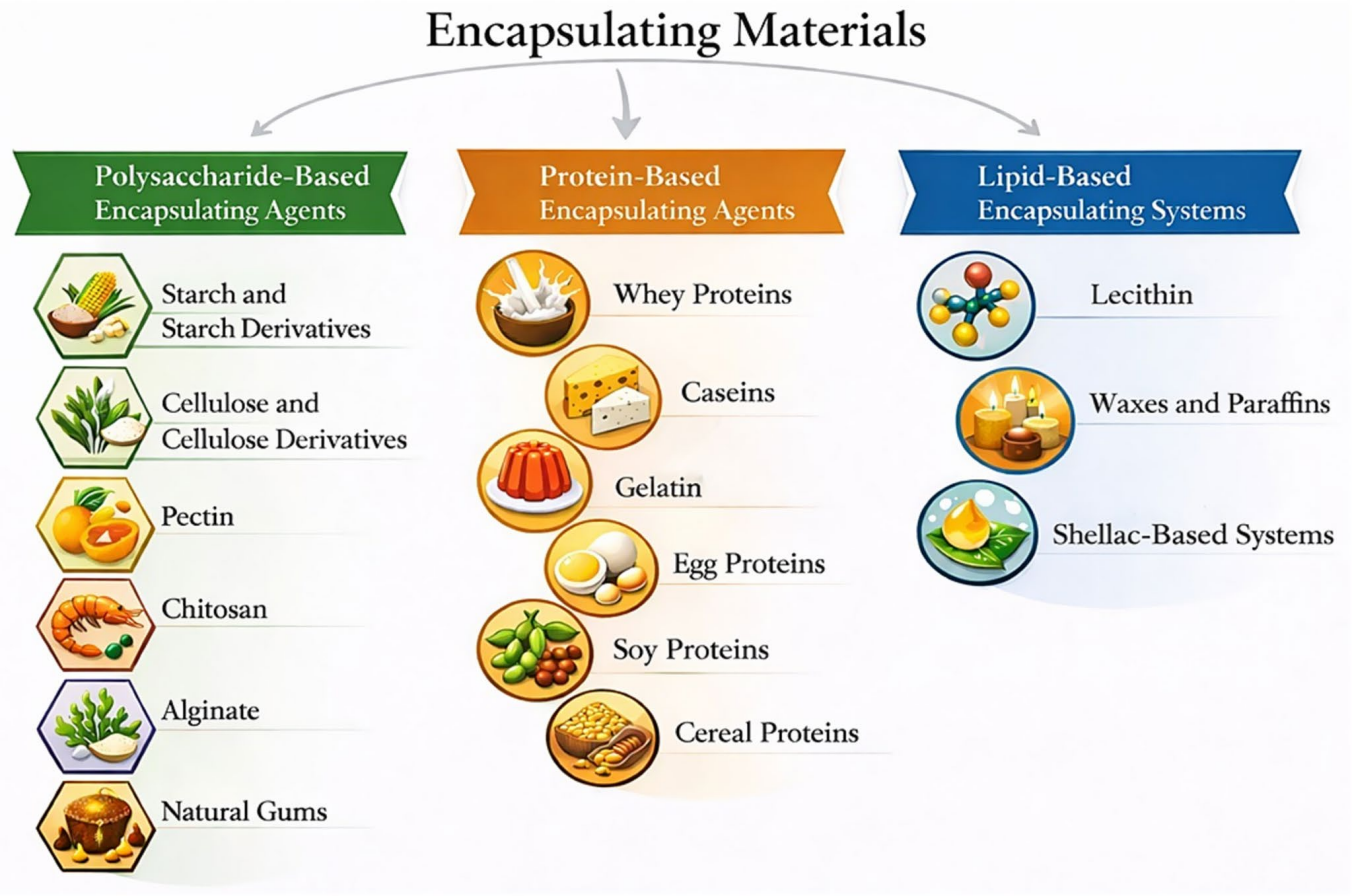
Comprehensive classification of encapsulating materials used in delivery systems.

Encapsulating materials play a crucial role in determining the efficiency, stability, and release behavior of bioactive delivery systems. The selection of suitable wall materials significantly influences encapsulation efficiency, protection against environmental stress, and the controlled release of bioactive compounds in food and nutraceutical applications. In recent years, extensive research has focused on developing natural, biodegradable, and food-grade encapsulating materials such as polysaccharides, proteins, lipids, and their composite systems to enhance the stability and bioavailability of sensitive bioactive molecules (1, 5). Polysaccharides, including starch, alginate, pectin, cellulose, and chitosan, are widely used due to their excellent film-forming ability, biocompatibility, and capability to form stable matrices for entrapping bioactive compounds (16, 27). Similarly, protein-based carriers such as whey proteins, caseins, gelatin, and plant proteins exhibit strong emulsifying, gelling, and binding properties, enabling efficient encapsulation and controlled release of both hydrophilic and hydrophobic bioactives (17, 18). Lipid-based carriers, including liposomes, nanoemulsions, and solid lipid nanoparticles, have also gained increasing attention because of their ability to improve the solubility, protection, and gastrointestinal bioaccessibility of poorly water-soluble compounds (12, 19). The structural diversity and functional characteristics of these encapsulating materials allow the development of tailored delivery systems for different classes of bioactive compounds. A comparative overview of commonly used encapsulating materials is summarized in Table 1. Figure 1 illustrates the major categories of encapsulating materials used for bioactive compound delivery systems. These materials provide the fundamental building blocks for designing efficient encapsulation systems that improve the stability, protection, and controlled release of functional bioactive ingredients in food and nutraceutical formulations.

**Table 1:** Comparative overview of encapsulating materials for bioactive compounds.

Material class	Specific material	Key functional properties	Best-suited bioactive compounds	Protection/mechanism	Sustainability/food-grade note	References
Polysaccharide	Starch/modified starch	Good film-forming and matrix-forming ability; tunable porosity; low allergenicity; low cost.	Hydrophilic & moderately hydrophobic polyphenols, pigments, vitamins.	Physical entrapment in nanoporous matrix; barrier to oxygen & light; controlled release via swelling/enzymatic degradation.	Widely food-grade and biodegradable; uses agricultural feedstocks; some nano-forms need safety assessment.	(16, 181, 182)
	Cellulose and derivatives	High mechanical strength, film forming, barrier (depending on derivative), modifiable surface chemistry.	Hydrophilic actives, probiotics (as scaffolds), and as stabilizer for hydrophobic payloads when combined with surfactants.	Physical protection via dense film/hydrogel; modified cellulose enables sustained release (diffusion control) and Pickering stabilization (nanocellulose).	Abundant, renewable; many derivatives are food-grade (CMC); biodegradability depends on modification.	(1, 27, 183)
	Pectin	Gelation with Ca^2+^ (LM pectin) or acid/heat (HM); mucoadhesive; hydrophilic networks.	Polyphenols, flavonoids, probiotic cells, hydrophilic vitamins.	Ionotropic gelation (alginate/pectin beads) and electrostatic interactions; protects against gastric conditions and enables colonic release	Fruit-derived, food-grade, biodegradable; degree of methylation affects release and enzymatic degradation in gut.	(15, 184)
	Chitosan	Cationic, mucoadhesive, antimicrobial activity; film-forming; pH-sensitive solubility (soluble in acidic pH).	Phenolics, peptides, probiotics (when layered), hydrophobic compounds combined with lipids.	Electrostatic complexation, polyelectrolyte coatings; pH-responsive release and improved mucosal adhesion.	Derived from chitin (shell waste); biodegradable and generally regarded as safe in many formulations, though regulatory acceptance varies by application.	(185, 186)
	Alginate	Rapid gelation with divalent cations; mild processing conditions; good for cell/probiotic encapsulation.	Probiotics, enzymes, hydrophilic vitamins, polyphenols (often co-encapsulated).	Ionotropic gelation producing hydrogel beads; physical barrier against acid and bile; can be layered/coated to reduce porosity.	Seaweed-derived, renewable, widely food-grade and used in foods; stability and release tuned via crosslinking and blends.	(69, 187)
	Natural gums	Emulsification, viscosity control, film-forming, stabilization of oil droplets.	Lipophilic bioactives in emulsions; flavors and hydrophilic actives in spray-drying.	Emulsion stabilization, film/coating formation, matrix entrapment after drying.	Plant/exudate sources, generally food-grade and biodegradable; supply sustainability varies (wild-harvested gums).	(188–190)
Protein	Whey proteins	Good emulsifying and gelling; heat-induced gelation and self-assembly; ligand-binding pockets for hydrophobic molecules.	Hydrophobic polyphenols, fat-soluble vitamins, peptides	Hydrophobic interactions + protein folding create core regions that bind hydrophobic actives; heat-induced networks/gel particles protect during storage and digestion.	Dairy-derived, widely food-grade; valorizes dairy by-products	(17, 191)
	Caseins/casein micelles	Natural micellar structure with amphiphilic domains; strong affinity for hydrophobic ligands; pH and Ca^2+^ responsive.	Strong for hydrophobic bioactives and small peptides.	Encapsulation via micellar entrapment (hydrophobic core), coacervation and re-assembly; provides protection from oxidation and gastric conditions (pH responsiveness).	Abundant dairy protein; GRAS use in foods; allergenicity noted.	(192–194)
	Gelatin	Thermo-reversible gelation, excellent film forming, good biocompatibility; easy chemical modification (crosslinking).	Peptides, probiotics (in matrices), hydrophilic and amphiphilic polyphenols; suitable for films and nanoparticles.	Gel matrix entrapment and film barriers; crosslinked gelatin slows release and improves stability against oxidation.	Animal-derived; food-grade but religious/ethical restrictions for some markets; fish gelatin offers alternative.	(18, 195)
	Egg proteins	Excellent foaming and gelation; fractionation yields functional peptides; good film-forming and nanoparticle potential.	Hydrophobic/hydrophilic small molecules, antimicrobials, peptides, probiotics.	Protein network formation and nanoparticles; protects actives and can confer antimicrobial properties (lysozyme).	Widely available, food-grade; allergenicity must be noted.	(77, 196)
	Soy proteins	Plant-based amphiphilic proteins; good emulsifying properties and capacity to form nanoparticles and Pickering stabilizers.	Lipophilic nutrients (DHA, carotenoids), plant pigments and polyphenols; ideal for vegan formulations.	Adsorption at oil–water interfaces (emulsions), nanoparticle matrices; complexation with polysaccharides for pH resistance.	Plant-based and sustainable alternative to animal proteins; generally GRAS.	(197, 198)
	Cereal proteins	Hydrophobic prolamins (zein) form strong nanoparticles/films; good barrier to moisture and oxygen when used as coatings.	Lipophilic bioactives (thymol, carotenoids), essential oils, hydrophobic nutraceuticals.	Hydrophobic matrix encapsulation (nanoprecipitation)—strong protection vs. oxidation; controlled release via erosion or enzymatic digestion.	Derived from agricultural residues (corn), renewable; often gluten/allergen concerns for some cereal proteins.	(80, 91)
Lipid	Lecithin	Amphiphilic phospholipids—natural emulsifiers; form liposomes, micro/nanoemulsions and contribute to oleogelation.	Lipophilic vitamins, carotenoids, essential oils, omega-3 fatty acids.	Interfacial stabilization; bilayer encapsulation protects from oxidation and improves bioavailability.	Widely used, food-grade; non-GMO/sunflower lecithin options exist for allergen-sensitive consumers.	(199, 200)
	Waxes and paraffins	Hydrophobic coatings with strong moisture barrier; good for fruit coatings/edible films; enable controlled volatile release.	Essential oils, volatile aroma compounds, lipophilic antioxidants applied as surface coatings or encapsulated in oleogels.	Hydrophobic barrier slows oxygen/moisture ingress, retards volatilization/oxidation; wax-based films embed actives in lipid matrix.	Natural waxes (carnauba, beeswax) are food-grade and biodegradable; paraffin (petroleum) less desirable for “natural” claims.	(201)
	Acetoglycerides	Potent food emulsifiers (low use levels), self-assemble into lyotropic phases useful for controlled release and oleogelation.	Lipophilic vitamins, omega-3 s, essential oils	Emulsification and self-assembled mesophases provide encapsulation and controlled diffusion; form structured lipids (oleogels) that trap actives.	Widely used food emulsifiers; enzymatic routes produce MAG/DAG from natural oils	(202, 203)
Resin/coating	Shellac-based systems	Excellent film formation, glossy coatings, good water-vapor barrier; edible insect-derived resin.	Surface protection for fruits; encapsulation/coating of polyphenols and hydrophobic actives for topical/fruit coatings.	Surface film/barrier; embedding of actives in shellac film slows oxidation/evaporation and provides mechanical protection.	Natural/semi-processed resin (from lac insect), food-grade when purified; ethical/supply considerations; biodegradable.	(204, 205)

### 2.1 Polysaccharide-based encapsulating agents

#### 2.1.1 Starch and starch derivatives

Starch is a naturally occurring polysaccharide composed of repeating glucose units and is widely distributed in food sources such as cereals, maize, potatoes, and rice. Structurally, starch granules are hydrophilic and consist of approximately 20–25% amylose and 75–80% amylopectin. Due to its biodegradability, wide availability, and relatively low cost, starch has been extensively utilized in various industrial and food applications (28). However, native starch exhibits limited emulsifying properties and predominantly hydrophilic characteristics, limiting its effectiveness in encapsulating hydrophobic bioactive compounds. To overcome these limitations, starch is often modified through chemical, enzymatic, physical, or biochemical treatments to alter its molecular structure and improve its functional properties for broader commercial applications (29). Depending on the physicochemical properties of the bioactive compound, encapsulation in starch matrices may occur via physical entrapment or chemical interactions. The high swelling capacity of starch enables bioactive molecules or extracts to be incorporated into the polymer matrix’s intermolecular network, where they may also function as plasticizing agents in starch-based films (30).

##### 2.1.1.1 Dextrins

Dextrins are water-soluble carbohydrates produced through the partial hydrolysis of starch and represent a group of modified starch derivatives characterized by different dextrose equivalent (DE) values. Certain types, such as brown and yellow dextrins, are highly water-soluble and are frequently used as carriers or diluents for food additives, including flavors, spices, and colorants. In particular, yellow dextrin has been used to encapsulate hydrophobic flavoring compounds and oils (29). When dextrins are generated through dry heat treatment, they are referred to as pyrodextrins. Based on their coloration, these are generally classified as white dextrin, yellow dextrin, and brown dextrin (also known as British gum) (31). Because dextrins are susceptible to enzymatic degradation by *α*-amylase, their chemical modification can be employed to tailor their structure for specific drug or bioactive delivery applications (32). Recent studies have also investigated the use of cluster dextrin to produce hydrolysate powders via spray drying, demonstrating that encapsulation with cluster dextrin can preserve antioxidant stability even after drying and simulated gastrointestinal digestion (33).

##### 2.1.1.2 Maltodextrins

Maltodextrin (MD) is produced by the controlled acid or enzymatic hydrolysis of starch and consists of glucose polymers linked through α-(1,4) and α-(1,6) glycosidic bonds. It is characterized by mild sweetness, high water solubility, and excellent dispersibility, yet exhibits limited solubility in alcohol (34). Maltodextrin is widely used as an encapsulating agent because it forms amorphous glassy structures that effectively entrap bioactive components. For example, freeze-drying of red wine (Cabernet Sauvignon) containing 20% (w/w) maltodextrin with DE10 removes water and most of the alcohol, while producing a stable amorphous matrix that retains the polyphenols present in the wine extract (35). In encapsulation systems, maltodextrin is frequently combined with other wall materials such as gums, pectin, alginate, and whey proteins to enhance emulsification properties, reduce oxygen permeability, improve bioactive retention, and regulate release kinetics (29). Additionally, encapsulation within maltodextrin matrices has been reported to significantly improve the stability of betalain pigments such as indicaxanthin derived from cactus pear. Under light-protected storage conditions at 20 °C, the encapsulated pigment demonstrated remarkable stability for several months, with no significant degradation or color changes (36).

##### 2.1.1.3 Cyclodextrins

Cyclodextrins are cyclic oligosaccharides produced through the enzymatic modification of starch. Their unique molecular structure resembles a truncated cone with a hydrophobic internal cavity and a hydrophilic outer surface. This configuration enables cyclodextrins to form inclusion complexes with poorly water-soluble compounds, such as polyphenols, thereby improving their aqueous solubility (29). Encapsulation using cyclodextrins has also been shown to significantly enhance the solubility of essential oils by up to 16 times, while simultaneously reducing photodegradation rates by up to 44 times and allowing the gradual release of the encapsulated compounds (37). The capacity of cyclodextrins to interact with a wide variety of organic molecules enables them to modify apparent solubility, increase resistance to heat-, light-, and oxidation-induced degradation, and reduce volatility. Owing to these advantageous properties, cyclodextrins have gained increasing importance in encapsulation and delivery applications (37).

#### 2.1.2 Cellulose and cellulose derivatives

Cellulose is the most abundant natural polysaccharide found in plant cell walls. Various chemically modified cellulose derivatives, including methylcellulose, carboxymethylcellulose, hydroxypropyl methylcellulose, and hydroxypropylcellulose, are widely used in encapsulation systems and in the production of edible films for food applications (28). Chemical modification of cellulose can also enhance its functional properties for delivery systems. For example, oxidation of cellulose beads using a TEMPO/NaClO₂/NaClO system significantly increased the anionic charge density to approximately 1.85 mmol g^−1^. Monitoring pH changes during the oxidation process revealed degradation of the oxoammonium ion, allowing optimization of reaction conditions to 48 h at 60 °C. Under these optimized conditions, the oxidized cellulose beads exhibited nearly double the drug release compared with non-modified reference beads, even at lower oxidation temperatures (38).

Cellulose has also been investigated as a carrier for phenolic compounds derived from raspberry juice. Freeze-dried cellulose–raspberry encapsulates were prepared to evaluate the influence of cellulose concentration (2.5, 5, 7.5, and 10%) and complexation time (15 or 60 min) on phenolic binding. The results indicated that formulations prepared with a lower cellulose concentration and a 15-min complexation period showed improved binding efficiency for phenolic compounds. These findings suggest that cellulose-based encapsulation systems can be developed under relatively simple, rapid processing conditions (39).

#### 2.1.3 Pectin

Pectin is a linear anionic polysaccharide primarily extracted from plant cell walls, particularly from citrus fruit peels. Structurally, it is mainly composed of *α*-(1 → 4)-linked D-galacturonic acid residues with varying degrees of methyl esterification and the presence of L-rhamnose units (40). Due to its natural origin, safety, and functional versatility, pectin is widely used in the development of delivery systems and nanostructured materials for encapsulating bioactive compounds. Pectin-based nanomaterials are commercially available and possess desirable physicochemical properties, making them suitable for various food and pharmaceutical applications.

Recent studies have shown that nanoencapsulation using pectin matrices can enhance the intestinal absorption and bioavailability of biologically active compounds (41). In addition to its technological applications, pectin consumption has been associated with several health benefits, including improved nutrient absorption, enhanced satiety, regulation of blood glucose levels, reduction of cholesterol, and positive modulation of the intestinal microbiota (42). Owing to these properties, pectin is frequently used in pharmaceutical formulations, particularly for the production of gastro-resistant capsules and colon-targeted drug delivery systems (43). The favorable physicochemical characteristics of pectin, combined with its relatively low cost and high encapsulation efficiency, have increased research interest in developing pectin-based nanostructures for controlled delivery of drugs and bioactive compounds within the gastrointestinal tract (44).

#### 2.1.4 Chitosan

Chitosan is a naturally derived, non-toxic polysaccharide widely employed in encapsulation systems due to its biocompatibility, biodegradability, and chemical stability. It also possesses excellent film-forming properties, often enabling film formation without the need for additional additives (45). Structurally, chitosan is a deacetylated derivative of chitin composed of N-acetyl-D-glucosamine units linked through β-(1 → 4) glycosidic bonds. In addition to its structural advantages, chitosan exhibits intrinsic antimicrobial and antioxidant activities, which further enhance its suitability for bioactive delivery systems (46).

Encapsulation studies have demonstrated the effectiveness of chitosan in stabilizing sensitive bioactive compounds. For instance, essential oil from *Lippia sidoides* was nanoencapsulated via spray drying using Angico gum and chitosan as wall materials, yielding nanoparticles ranging from 10 to 60 nm with an encapsulation efficiency of approximately 77.8%. These nanoparticles exhibited strong larvicidal activity against *Aedes aegypti*, the mosquito vector of dengue fever (47). Chitosan is also considered beneficial for dietary applications due to its ability to bind lipids in the gastrointestinal tract, making it a promising ingredient for weight-management foods (48).

Furthermore, chitosan nanoparticles have been reported to enhance the stability of tea polyphenols by protecting them from oxidation and degradation during gastrointestinal digestion, thereby improving their absorption in intestinal epithelial cells (49). Because chitosan carries positive charges from its amino groups, it can readily interact with negatively charged polymers and crosslinking agents to form stable complexes. For example, crosslinking chitosan with cellulose nanocrystals has been shown to produce stable microcapsules with improved anthocyanin encapsulation efficiency and enhanced stability (50). Chitosan has also been used as a co-encapsulating agent for bioactive compounds such as curcumin and resveratrol, as well as in nanocomposite films designed to inhibit the growth of fungal pathogens, including *Penicillium chrysogenum*, *Aspergillus flavus*, *Aspergillus niger*, and *Aspergillus parasiticus* (51, 52).

#### 2.1.5 Alginate

Alginate is a naturally occurring hydrophilic polysaccharide obtained from brown seaweed and is widely applied in food systems due to its gelling, thickening, stabilizing, and film-forming capabilities (45). Owing to these functional properties, alginate has been extensively utilized as a carrier material for the delivery of drugs and bioactive compounds in food, medical, and pharmaceutical applications (53). Chemically, alginate is composed of β-D-mannuronic acid (M) and *α*-L-guluronic acid (G) units linked through glycosidic bonds. These monomeric units are arranged in different block structures, including homopolymeric M-blocks, homopolymeric G-blocks, and alternating MG-blocks along the polymer chain (54).

The ratio of M and G residues strongly influences the physicochemical characteristics of alginate gels. Alginate containing higher proportions of G-blocks tends to form stronger, more rigid hydrogels with larger pores, facilitating the diffusion of entrapped bioactive compounds (55). In contrast, alginate rich in M-blocks forms softer, more flexible matrices suitable for edible coatings and capsules with lower permeability (56). In encapsulation applications, sodium alginate and calcium-alginate systems are particularly favored due to their ease of preparation, low cost, non-toxicity, and excellent biocompatibility (29). In addition to ionic crosslinking, alginate also exhibits strong pH sensitivity. Variations in pH significantly influence the swelling behavior of alginate gels and the release kinetics of encapsulated bioactive compounds during gastrointestinal digestion, making alginate an ideal material for controlled release systems (57).

#### 2.1.6 Natural gums

Natural plant-derived gums are commonly categorized based on their source, including seed gums (e.g., guar gum), plant exudate gums (e.g., gum arabic), microbial gums (e.g., xanthan gum), and seaweed-derived gums (e.g., carrageenan) (29). These polysaccharides are water-soluble biopolymers that typically form viscous colloidal dispersions when hydrated. Due to their high molecular weight and strong intermolecular interactions, many seed gums are generally tasteless, odorless, and colorless, but they do not form true molecular solutions (58). Natural gums have been widely explored as encapsulating agents due to their ability to stabilize emulsions and improve the retention of bioactive compounds. Incorporating gums into encapsulation systems has been shown to enhance the encapsulation efficiency of polyphenols such as quercetin, while also improving antioxidant activity and physical stability of the resulting delivery systems (59).

### 2.2 Protein-based encapsulating agents

#### 2.2.1 Whey proteins

Whey proteins are widely recognized for their excellent functional and biological properties, particularly their strong gelling and emulsifying capabilities. These proteins are frequently used in the preparation of hydrogels, nanoparticle-based carriers, and complex coacervate systems formed with various polysaccharides (29). Among the whey protein components, *β*-lactoglobulin (BLG) is the predominant fraction and serves as the primary contributor to gel formation. Due to their distinctive physicochemical characteristics, whey proteins have attracted significant interest for both food and non-food applications. For instance, whey protein gels have been explored as pH-responsive hydrogel matrices for the controlled delivery of biologically active compounds (60).

Whey protein–based vehicles (WPVs) offer versatile structural configurations that can be tailored for specific delivery applications due to their diverse functional properties (61). Hydrogels are, in general, three-dimensional polymeric networks capable of absorbing and retaining large amounts of water. This water-holding capacity arises from the presence of hydrophilic functional groups such as –OH, –CONH–, –CONH₂, –COOH, and –SO₃H, which interact strongly with water molecules. When used as encapsulating matrices, WPVs—including micro- and nanoparticles, hydrogels, nanogels, emulsions, Pickering emulsions, conjugates, complexes, and nanotubular structures—can enhance the bioavailability of bioactive compounds while protecting them from environmental stresses such as light exposure, oxygen, temperature fluctuations, and enzymatic degradation. Furthermore, these delivery systems enable sustained or controlled release of encapsulated compounds. Importantly, whey protein-based carriers are biodegradable and typically degrade via natural biological processes within the body (62, 63).

#### 2.2.2 Caseins

Casein represents a group of milk proteins composed mainly of four major fractions: αs1-casein, αs2-casein, β-casein, and *κ*-casein. Although these proteins exhibit similar molecular weights of approximately 24 kDa, they differ significantly in amino acid composition and in their affinity toward hydrophilic and hydrophobic compounds (64). Owing to these structural characteristics, caseins are considered effective natural carriers for the encapsulation of bioactive molecules, particularly hydrophobic compounds such as β-carotene (65), quercetin derived from onion peel (66), and resveratrol (67).

Casein molecules readily interact with calcium ions to form organized spherical aggregates known as casein micelles. These micellar structures typically range from 50 to 500 nm in diameter, with an average size of approximately 150 nm, and consist of about 94% protein and 6% minerals, including calcium, phosphate, magnesium, and citrate (68). The use of casein micelles as delivery vehicles offers several advantages, such as improved processing stability of encapsulated bioactive compounds and reduced production costs compared with synthetic emulsification systems. Additionally, casein-based carriers are considered sustainable and environmentally friendly encapsulation systems because they can be produced without organic solvents (69).

#### 2.2.3 Gelatin

Gelatin is a protein obtained from animal-derived collagen through various hydrolysis processes, including thermal, enzymatic, acidic, or alkaline treatments (70). Collagen, the most abundant structural protein in mammals, is characterized by its unique triple-helix structure, which provides remarkable mechanical strength and structural stability (71). Commercially, gelatin is classified into two major types based on the extraction method. Gelatin type A is typically derived from bovine, porcine, or fish skin through acid hydrolysis, whereas gelatin type B is produced from bones using alkaline hydrolysis (72).

Gelatin has been extensively investigated as a carrier matrix for encapsulating bioactive compounds. For example, cinnamon oil emulsions incorporated into gelatin films at a 70:30 ratio significantly improved the mechanical properties, water barrier characteristics, antioxidant activity, and thermal stability of the films. The enhanced antioxidant activity indicated that the gelatin matrix effectively preserved the stability of encapsulated cinnamon oil, highlighting its potential use in active food packaging applications (73). In another study, camel skin gelatin (CSG) combined with camel casein (CC) was used to encapsulate probiotic strains, including *Lactobacillus rhamnosus*, *Pediococcus pentosaceus*, and *Lactobacillus paracasei*. The encapsulated probiotics demonstrated significantly improved survival during simulated gastrointestinal digestion compared with free cells. Moreover, their thermal resistance at 50 °C and 70 °C was considerably enhanced when encapsulated within CC and CC–CSG matrices (74).

Despite these advantages, gelatin has certain limitations when used alone in food packaging applications. Due to their hygroscopic nature and strong intermolecular interactions, gelatin films tend to be brittle and prone to cracking, thereby limiting their mechanical durability (75).

#### 2.2.4 Egg proteins

Hen eggs contain approximately 12% protein by weight, primarily distributed between the albumen (egg white) and yolk in proportions of roughly 44 and 50%, respectively (76). Both egg white and yolk contain a diverse range of proteins with distinct structural and physicochemical characteristics, including the ability to form gels, self-assemble, and modify surfaces. These properties make egg proteins highly adaptable for the design of various bioactive delivery systems (77, 78). Egg white proteins consist of several major components, including ovalbumin (54%), ovotransferrin (12%), ovomucoid (11%), lysozyme (3.5%), and ovomucin (3.5%), along with minor proteins such as G2 globulin, G3 globulin, ovoinhibitor, ovoglycoprotein, ovoflavoprotein, ovomacroglobulin, avidin, and cystatin (79). Currently, proteins such as ovalbumin, lysozyme, and whole egg white protein are most commonly used in delivery systems, whereas other proteins, such as ovotransferrin, ovomucoid, and ovomucin, remain relatively underexplored (80). Research by Yao et al. (81) demonstrated that ethanol-induced gelation of egg white proteins can form stable matrices capable of encapsulating tea polyphenols and curcumin, thereby improving their stability, antioxidant activity, and bioavailability. Gel formation was associated with higher absolute zeta potentials and higher levels of free sulfhydryl groups. Egg white proteins are also capable of binding polyphenolic compounds, making them promising carriers for delivering bioactive molecules. Consequently, compounds such as curcumin (82) and anthocyanins (244) are often employed as model bioactives to evaluate egg protein-based delivery systems.

#### 2.2.5 Soy proteins

Soy proteins are widely considered suitable encapsulating agents because of their good water solubility, strong adsorption at oil–water interfaces, and favorable gel-forming and film-forming properties (83). These functional attributes enable soy proteins to effectively encapsulate hydrophobic compounds, including palm oil, rambutan seed oil, sunflower oil, paprika oleoresin, and palm stearin (84, 85). Soy protein isolate (SPI) is a high-quality plant-derived protein primarily composed of 7S and 11S globulins, which are capable of self-assembly into ordered nanostructures (86). When SPI is combined with polysaccharides, the resulting composite matrices exhibit improved oxidative stability, encapsulation efficiency, and drying characteristics (87). In a recent study, a nanocarrier system composed of SPI and fucoidan (Fuc) was developed to encapsulate curcumin through electrostatic interactions under both acidic and neutral conditions. The resulting SPI–Fuc nanoparticles displayed a spherical core–shell structure with an average particle size of approximately 236.56 nm and achieved an encapsulation efficiency exceeding 95%. Additionally, the nanoparticle system exhibited excellent dispersion stability over extended storage periods (88).

#### 2.2.6 Cereal proteins

##### 2.2.6.1 Zein

Zein is a major storage protein derived from maize and belongs to the prolamin family of cereal proteins. It consists primarily of four fractions: *α*-, *β*-, *γ*-, and *δ*-zein. This protein is characterized by its water-insolubility, biodegradability, and biocompatibility, along with a strong tendency for molecular self-assembly, which enables the formation of diverse nanostructures across different solvent systems. In addition, zein possesses inherent hydrophobic characteristics that facilitate the encapsulation of lipophilic compounds (29). Although other plant proteins, such as soy and pea proteins, are widely used in functional foods and biomedical formulations, zein stands out for its structural stability, hydrophobic nature, and high loading capacity for hydrophobic bioactive compounds (89). Consequently, zein has gained considerable attention as a promising carrier material for the development of delivery systems in food and pharmaceutical applications. Studies have also shown that bioactive compounds co-encapsulated within zein matrices may exhibit synergistic biological effects and enable sustained and controlled release under food system conditions (90). Zein-based nanoparticles have been successfully employed to encapsulate a wide range of active ingredients, including antioxidants, antimicrobial compounds, and essential oils. These nanocarriers can gradually release the encapsulated compounds, thereby protecting food systems from oxidative deterioration, microbial contamination, and spoilage, ultimately contributing to extended shelf life (91).

##### 2.2.6.2 Wheat protein

Wheat gluten is a protein complex obtained as a by-product during the separation of starch from wheat flour. It is composed mainly of proteins with a minor proportion of polysaccharides and consists of two principal fractions: gliadin and glutenin. Gliadin is a single-chain polypeptide with a molecular weight of approximately 25–100 kDa. It is soluble in neutral 70% ethanol and stabilized by intramolecular disulfide bonds. In contrast, glutenin has a molecular mass exceeding 105 kDa and forms large polymeric aggregates through intermolecular disulfide linkages among gliadin-like subunits (92). Wheat proteins have been successfully used as encapsulation matrices, either alone or in combination with polysaccharides, to improve the stability and delivery of bioactive compounds. However, gluten proteins are known allergens and are associated with celiac disease in sensitive individuals (29). A recent investigation by Jiabao reported that a wheat gluten protein matrix combined with pterostilbene (WGPM-PTE) demonstrated superior encapsulation efficiency. Scanning electron microscopy further revealed a smoother surface morphology and a more compact gel-like network structure compared with other tested systems (93).

##### 2.2.6.3 Barley protein

Proteins in barley are largely dominated by hordeins, which account for approximately 30–50% of the total protein fraction. Hordeins belong to the prolamin class of proteins and are rich in the amino acids glutamine and proline. Rather than existing as a single protein, hordeins represent a heterogeneous group of polypeptides with varying structures and properties. The remaining protein fraction in barley mainly consists of albumins, globulins, and glutelins (94). Based on their amino acid composition and extraction characteristics, hordeins can be further subdivided into several types, among which B-hordein is the predominant fraction, representing around 70–90% of the total hordein content and serving as the primary storage protein in barley grains (95). Due to their functional attributes—such as emulsifying ability, foam stabilization, elasticity, and cohesive properties—barley proteins are considered promising materials for the fabrication of encapsulation microparticles (96). Early research by Wang and colleagues demonstrated the potential of barley protein as a carrier matrix for encapsulating fish oil. The resulting microcapsules showed high encapsulation efficiency and loading capacity while effectively protecting fish oil from oxidative degradation, indicating their suitability for incorporation into food systems.

##### 2.2.6.4 Rice protein

The protein content of white rice generally ranges from 6.4 to 14.8%, which is relatively lower than that of many other cereal grains. Among the different protein fractions in rice, glutelin is the major component, followed by albumin, globulin, and prolamin (97). Rice proteins have been investigated as potential carrier materials for delivering bioactive compounds due to their favorable digestibility and relatively high biological value compared with other cereal proteins (98). For example, brown rice proteins have been explored as delivery systems for raspberry-derived phenolic compounds and volatile components. In another study, Kopjar and co-workers evaluated brown rice protein and almond protein matrices for the encapsulation of quercetin. The findings indicated that the brown rice protein matrix exhibited a stronger affinity for quercetin, suggesting that it could serve as a more efficient carrier for this phenolic compound. Furthermore, increasing the quercetin concentration in the initial formulation resulted in a proportional increase in the amount of quercetin incorporated into the microparticles (99).

##### 2.2.6.5 Amaranth protein

Amaranth (Amaranthus spp.) is an underutilized pseudocereal, classified as a C4 crop, with a relatively high protein content of 13 to 22%. The protein fraction is mainly composed of albumins and globulins (100). Recently, amaranth proteins have attracted attention for the development of nanocarrier systems designed for the delivery of bioactive compounds. Proteins possess distinct structural and functional properties that make them highly suitable for the construction of encapsulation systems (101). Amaranth proteins, in particular, exhibit excellent gel-forming properties, enabling the formation of gel-based structures such as nanogels and microgels. In addition, they contain a balanced profile of essential amino acids, enhancing their nutritional quality (77, 78). In one study, amaranth protein was blended with the carbohydrate polymer pullulan in formic acid to produce fibrous structures. The formation of well-defined fibers with higher protein content was achieved only after the addition of a surfactant. Subsequent work incorporated curcumin into the fiber matrix, and the encapsulated compound retained its antioxidant activity following simulated gastrointestinal digestion (102).

### 2.3 Lipid-based encapsulating systems

#### 2.3.1 Lecithin

Lecithin is one of the most widely used lipid-based encapsulating materials due to its amphiphilic nature, which enables the formation of stable emulsions, liposomes, and lipid vesicles for the delivery of hydrophobic and hydrophilic bioactive compounds (103). Structurally, lecithin is composed mainly of phospholipids such as phosphatidylcholine, phosphatidylethanolamine, and phosphatidylinositol, which possess both hydrophilic head groups and hydrophobic fatty acid tails. This dual polarity facilitates the spontaneous formation of bilayer structures that can entrap bioactive molecules within lipid vesicles or liposomal carriers (104). In food and nutraceutical systems, lecithin-based encapsulation has been extensively employed for the protection and controlled delivery of sensitive compounds such as curcumin, polyphenols, vitamins, carotenoids, and essential oils (105). Recent studies have demonstrated that lecithin-based liposomes significantly improve the stability, solubility, and bioavailability of poorly water-soluble bioactive compounds. For instance, phospholipid liposomes have been successfully used to encapsulate curcumin and resveratrol, enhancing their oxidative stability and gastrointestinal bioaccessibility (106). Moreover, lecithin nanoparticles and nanoliposomes can protect bioactives against environmental stresses such as oxidation, light exposure, and enzymatic degradation during food processing and storage (107). The combination of lecithin with biopolymers such as chitosan or alginate has further improved encapsulation efficiency and controlled release properties. Due to its natural origin, GRAS status, and excellent emulsifying properties, lecithin remains a key material for the design of lipid-based delivery systems in functional foods and nutraceutical applications (108).

#### 2.3.2 Waxes and paraffins

Waxes and paraffins are hydrophobic lipid materials frequently utilized as coating agents for the encapsulation of bioactive compounds that require protection against moisture, oxygen, and thermal degradation (109). Natural waxes such as beeswax, carnauba wax, and candelilla wax, as well as petroleum-derived paraffins, possess high melting points and low permeability to water vapor and gases, making them suitable matrices for controlled release systems (110). The hydrophobic structure of wax matrices enables effective encapsulation of lipophilic compounds, including essential oils, flavors, fat-soluble vitamins, and antioxidants. Recent advances have shown that wax-based microcapsules can significantly enhance the oxidative stability and shelf life of encapsulated ingredients by creating a physical barrier against environmental factors (111). Additionally, wax-based systems exhibit slow, controlled release characteristics due to their crystalline structure, which gradually melts or degrades under specific conditions, such as temperature changes or mechanical stress (112). Studies have also explored incorporating waxes into hybrid encapsulation systems with proteins or polysaccharides to improve encapsulation efficiency and functional performance (113). Consequently, wax- and paraffin-based carriers have gained increasing attention for applications in food preservation, nutraceutical delivery, and the stabilization of functional ingredients.

#### 2.3.3 Shellac-based systems

Shellac is a natural resin secreted by the insect *Kerria lacca* and has been widely used as a coating and encapsulating material in pharmaceutical, food, and nutraceutical industries. It possesses excellent film-forming ability, low permeability to gases and moisture, and high resistance to acidic environments (114, 115). These characteristics make shellac particularly suitable for developing pH-sensitive encapsulation systems that protect bioactive compounds during gastric digestion while enabling targeted release in the intestinal environment (116). Shellac-based matrices effectively protect sensitive bioactive compounds, such as probiotics, polyphenols, vitamins, and essential oils, from degradation caused by oxygen, heat, and light (117, 118). Recent studies have demonstrated that shellac nanoparticles can significantly enhance the stability and controlled release of nutraceutical compounds during gastrointestinal digestion (117). Furthermore, shellac is often combined with other biopolymers such as proteins, polysaccharides, or lipids to develop composite encapsulation systems with improved mechanical strength and functional performance (119). Due to its natural origin, biodegradability, and excellent barrier properties, shellac-based encapsulation systems have gained increasing interest for targeted delivery and controlled release applications in functional foods and nutraceutical products.

#### 2.3.4 Medium and short-chain triglycerides

Medium-chain triglycerides (MCTs) and short-chain triglycerides (SCTs) have emerged as highly effective lipid carriers for encapsulating hydrophobic bioactive compounds due to their unique physicochemical and metabolic properties. MCTs, typically composed of C6–C12 fatty acids, exhibit high solubilization capacity, rapid digestibility, and excellent oxidative stability, making them particularly suitable for lipid-based delivery systems such as nanoemulsions, solid lipid nanoparticles (SLNs), nanostructured lipid carriers (NLCs), and oleogels (120). The development of curcumin-loaded nanostructured lipid carriers formulated with blends of cocoa butter and MCT oil (0–10 wt%). These systems demonstrated excellent physicochemical stability under a wide range of processing and environmental conditions, including temperatures from 25 to 121 °C, ionic strengths of 50–500 mM, and pH values of 2.0–6.0. Importantly, the inclusion of MCTs reduced lipid crystallinity, thereby increasing drug loading capacity and improving supersaturation levels (1863–2,328%), which were significantly higher than conventional nanoemulsions (1,489 ± 6%). Furthermore, the MCT-based carriers achieved enhanced bioaccessibility of curcumin (79.4–91.1%) and controlled release behavior, demonstrating their effectiveness in improving the delivery of poorly water-soluble compounds (121). Similarly, structured lipid systems incorporating both medium- and long-chain triglycerides (MLCTs) have been successfully used for encapsulation of *ω*-3 fatty acids. MLCT microcapsules prepared using protein-based wall materials (e.g., soy protein) achieved very high encapsulation efficiency (94.56%) and exhibited excellent oxidative stability and controlled release properties (122). The small particle size, low polydispersity, and favorable interfacial characteristics contributed to the structural integrity of the microcapsules during spray drying and storage. These systems demonstrated strong potential as carriers for lipophilic nutraceuticals, owing to their enhanced stability and sustained release.

MCT-based oleogel systems have also gained attention as alternative encapsulation matrices for bioactive compounds such as β-carotene. Beeswax–MCT oleogels containing β-carotene (122–124 μg/100 g) exhibited improved thermal stability, with melting temperatures increasing from 51.5 °C to 55.2 °C as the gelator concentration increased (5–15%). These systems also showed high oxidative stability and enhanced smoke points (246–261 °C), making them suitable for functional food applications involving high-temperature processing. The structured network formed by MCTs not only protects the encapsulated compound but also improves texture and shelf-life stability, highlighting their applicability in both food and cosmetic formulations (123). Short-chain triglycerides, although less commonly studied than MCTs, also contribute to encapsulation systems by modifying lipid matrix properties such as polarity, crystallinity, and release kinetics. When combined with MCTs or long-chain lipids, they help tailor the internal structure of lipid carriers, facilitating improved loading and controlled release of active compounds. Recent reviews emphasize that triglyceride-based systems (including SCTs and MCTs) play a crucial role in the design of advanced delivery systems, such as emulsions, oleogels, and hybrid lipid carriers, which enhance the bioavailability and functional performance of encapsulated bioactives (105). Recent studies demonstrate that MCT- and SCT-based encapsulation systems significantly improve the stability, solubility, and bioaccessibility of bioactive compounds. Their ability to modulate lipid matrix structure, enhance encapsulation efficiency (>80–95% in many systems), and provide controlled release makes them highly promising materials for applications in food, nutraceutical, and pharmaceutical industries.

## 3 Advanced encapsulation techniques

The section on advanced encapsulation techniques provides a comprehensive overview of modern approaches designed to improve the stability, protection, and delivery efficiency of bioactive compounds in food and nutraceutical systems. These techniques integrate principles of material science, food engineering, and nanotechnology to overcome the inherent limitations of conventional encapsulation methods, such as low encapsulation efficiency, poor control over release kinetics, and instability under processing and storage conditions. The major characteristics, advantages, and disadvantages of each encapsulation technology are mentioned in Table 2. The following subsections discuss key advanced encapsulation methods in detail, highlighting their working principles, recent advancements, and applications in food and nutraceutical systems. Further, encapsulation of bioactive compounds using advanced encapsulation techniques has been reported in Table 3.

**Table 2:** Key characteristics, advantages and disadvantages of each encapsulation techniques.

Encapsulation technique	Key characteristics	Advantages	Disadvantages	References
Ionic gelation	Ion-induced crosslinking of biopolymers forming hydrogels	Mild conditions, cost-effective, suitable for heat-sensitive compounds, easy processing	Poor mechanical strength, limited scalability, possible burst release	(206)
Electrospinning	Formation of nanofibers using electric field	High surface area, controlled release, high encapsulation efficiency	Requires specialized equipment, solvent limitations, scale-up challenges	(137)
Complex Coacervation	Phase separation via electrostatic interaction between polymers	High encapsulation efficiency, good protection of sensitive compounds, tunable release	Sensitive to pH and ionic strength, process complexity, stability issues	(207)
Liposome-assisted encapsulation	Phospholipid bilayer vesicles encapsulating hydrophilic/lipophilic compounds	Improves bioavailability, biocompatible, suitable for targeted delivery	Low physical stability, high cost, potential leakage during storage	(11)
Fluidized bed coating	Layer-by-layer coating of particles using fluidization	Enhanced stability, controlled release, scalable	Requires pre-formed particles, energy-intensive, coating uniformity issues	(208)
High-pressure homogenization (HPH)	Formation of nanoemulsions via high shear forces	Produces small particle size, improves stability and bioavailability	High energy input, possible degradation of sensitive compounds	(209)
Extrusion–spheronization	Formation of spherical pellets through extrusion and rounding	Uniform particle size, good control over release	Limited to specific materials, multi-step process, lower encapsulation efficiency for liquids	(210)

**Table 3:** Recent advances in encapsulation of bioactive compounds using advance encapsulation technique.

Encapsulation techniques	Coating materials	Core compounds	Particle size	Encapsulation efficiency (%)	Purpose/application	References
Ionic gelation	Sodium Alginate	Propolis	-	87	Food Application	(128)
	Sodium Alginate	Lemon Balm Extract	3.96 nm	-	Preservation of aroma and volatile compounds	(129)
	Pectin	Pitanga extract	455 to 676 μm	54.51	Application in foods as a natural colorant and/or functional agent	(130)
	Sodium Alginate	Papaya leaf extract	3.66 μm	85.6	Functional food applications	(131)
	Sodium Alginate	*Moringa oleifera* seed extract	4.58 mm	75.37	Preservation of Extract	(132)
	Alginate, pectin, whey protein	Blueberry Extracts	-	74.55	Protecting the health promoting compounds	(211)
	Sodium alginate, maltodextrin	Green tea extracts	1,399–2034 nm	86.86	Fortification in functional food products	(212)
	Sodium alginate	Protein hydrolysate from Lionfish muscle proteins	2–3 mm	55.47	Functional food application	(213)
	Sodium alginate	Purple tea polyphenols	-	84	Application in Tea	(214)
	Sodium alginate	Grape Pomace Extract	-	56.25	Functional ingredient in products for oral administration	(134)
	Pectin, corn starch	Pomegranate polyphenols	2.90–3.32 mm	42–101	Functional food application	(215)
	Chitosan, alginate	*Moringa oleifera* leaf extract	-	95.02	Applications if food and pharmaceuticals	(133)
Electrospinning	Zein	Grape pomace extract	-	-	Potential uses in food and pharmaceutical products	(138)
	Gelatin, OSA-starch	Sage Extract	231.62 nm	87.5	Delivery systems for bioactive compounds in functional foods	(139)
	Gelatin, cyclodextrin	Eugenol and Thymol	190 and 160 nm	-	Rapid oral delivery of natural bioactives	(216)
	Potato protein, pullulans	Blueberry extract	200–300 nm	50	Potential alternative to animal proteins for creating nanostructures	(217)
	Whey protein isolate	Omega-3	262.7 nm	97.6	Encapsulate bioactive compounds used in functional food products	(140)
	Polyvinyl alcohol, fucoidan	*Lactiplantibacillus plantarum* and polyphenols	338.04 nm	-	Developing novel functional foods with higher probiotics	(218)
	Zein	Carotenoid	304 nm	77.78	Nanofibers changed color of the foods during the storage	(141)
	Gum Arabic, whey protein isolate	β-carotene	73–96 nm	>90	Potential for producing nanostructurs loaded with liposoluble drugs/bioactives	(219)
	Zein	Cumin essential oil	459 to 855 nm	nearly 100	Active packaging application for various foods such as cheese, meat and some other food products	(220)
Complex coacervation	Soy protein and inulin	Bioactive compounds from tender aromatic coconut mesocarp	385.90 nm	98.74	Protecting phenolic compounds during digestion and enhancing their potential bioavailability	(143)
	Whey protein, gum Arabic	Propolis	3.14 μm	80.3	Potential of encapsulated propolis extract as a food additive	(144)
	Gelatin, pectin	Bioactive compounds from red onion skin	-	80	Coacervation provides a sustained delivery of the compounds into nutraceutical food	(221)
	Chitosan, gum Arabic	Saffron petal and *Stachys schtschegleevii* extracts	382.32 and 579.06 nm	89.85 and 84.52	Co-encapsulation system improved bioavailability through the co-delivery of core materials	(145)
	Maltodextrin, whey protein isolate, gum Arabic	Black carrot extract	-	86.08	Provide valuable insights into the stability and release dynamics in different food settings	(222)
	Gelatin, gum acacia	Anthocyanin from black rice bran			could be utilized as a potential source of stable nutraceutical	(223)
	Gelatin, carrageenan	*Citrus limon* essential oil	-	93	Microcapsules can safeguard essential oils composition, potentiating their exploitation as functional food ingredients	(146)
	Wheat germ protein, high methoxy pectin	d-limonene	623.37 nm	-	Effective delivery systems for volatile aromas or bioactive compounds	(224)
	soy protein isolate, sodium casinate, sodium carboxymethylcellulose, sodium alginate	Green coffee oil	72.57–295.00 μm	90.01	Potential use of GCO in the development of powder food	(225)
	Gelatin, sodium alginate	Propolis	-	91.86	Propolis microcapsule can be used as a healthy drink product in the food industry	(226)
	Zein, potato starch	*Rosa damascena* mill L. anthocyanin	50–175 nm	89.36	Suitable for drug delivery processes	(227)
Liposome	Lecithin	Propolis Extract	0.443 μm	76.12	Apple juice enriched with these liposomes increased the stability and preservation of bioactive compounds during digestion	(147)
	Lecithin	*Inonotus obliquus* (Chaga) Extracts	175 nm	-	Enhance the stability and practical applicability of Chaga antioxidants in nutraceutical or therapeutic contexts	(228)
	Lecithin, cholesterol	Mangiferin	129.53 nm	70.63	Potential applications in functional foods and nutraceuticals	(148)
	Lecithin, β-sitosterol	*Centella asiatica* polyphenols	700 nm	74.789	Suitable for functional food applications	(107)
	Unsaturated phospholipid, cholesterol	Olive leaves and orange peels	96 and 101 nm, respectively	29 and 11, respectively	Enhanced antibacterial activity against *S. aureus*	(229)
	Unsaturated phospholipid, cholesterol	*Olea europaea* Leaf Polyphenols	79–120 nm	72	Increased antimicrobial activity compared to the free extracts	(230)
	Lecithin, stigmasterol	*Centella asiatica* leaf extract	787.78 nm	67.80	Utilised to generate new fortified food products with health benefits	(104)
	Lecithin, cholesterol	Garlic essential oil	159.8 nm	95	Enrich different foods, including dairy products and beverages	(231)
Fluidized bed	Zein	Grape Skin Extract	120.13 μm	98.98	Valorizing grape by-products as functional food ingredients	(150)
	Maltodextrin, whey protein and hydroxypropyl methylcellulose	Linseed Oil	19.32 μm	63.5	Used in special low-calorie diets	(151)
	Maltodextrin	V B12	559 μm	99.6	Developing oral fortified foods	(232)
	Pectin	Yerba Mate Extract	-	-	Application in fruit and cereal bars is feasible.	(233)
	Maltodextrin	*Spirulina platensis*	-	-	Yoghurt fortification	(152)
	Egg albumin	Betalains	167.1 μm	-	Bioactive stabilization	(234)
	Medium-chain triglycerides	Date pit Phenolic Compounds	-	-	Application in bakery product to extend the shelflife	(235)
High pressure homogenization	Maltodextrin, Arabic gum, Sodium alginate	Polyunsaturated Fatty Acids	369.4 nm	95.6	Extend the oxidative stability	(236)
	Chitosan	Astaxanthin	109.97 nm	90.28	Extend the oxidative stability	(153)
	sunflower lecithin, carboxymethylcellulose sodium salt	Anthocyanins	165–405 nm	84.61	Natural dye application	(154)
	Camellia seed oil, Chitosan	Curcumin	280.87 nm	~99	Functional food products development	(237)
	Pterostilbene	Hydroxypropyl-β-cyclodextrin	-	94.34	Improved the stability and bioaccessibility	(155)
	Cellulose nanofibrils	Fucoxanthin	87–295 nm	70	Development of drug	(238)
	β-cyclodextrin, decapolyglycerol monooleate, soybean lecithin	Astaxanthin	206.9 nm	99.65	Application in food and health products	(239)
	Sodium starch octenylsuccinate and inulin	Paprika Oleoresin	255–901.7 nm	85.47–99	Functional food applications	(77)
Extrusion-spheronization	Poly(3-hydroxybutyrate-*co*-3-hydroxyvalerate)	Cinnamic acid (CA)	-	-	Inhibition of *Phytophthora*	(240)
	Microcrystalline cellulose, isomalt, and crospovidone	Cilostazol	252 nm	-	Increase the in vitro solubility and dissolution	(241)
	Hydroxypropyl methylcellulose, Eudragit® RL	Drug-containing pellet	-	96.6	Applications of chronotherapy with drugs are expected	(242)
	Microcrystalline cellulose, hydroxypropylmethylcellulose	Glipizide	600–900 μm	-	Design of drug delivery system	(243)

### 3.1 Ionic gelation

**Figure 2:**
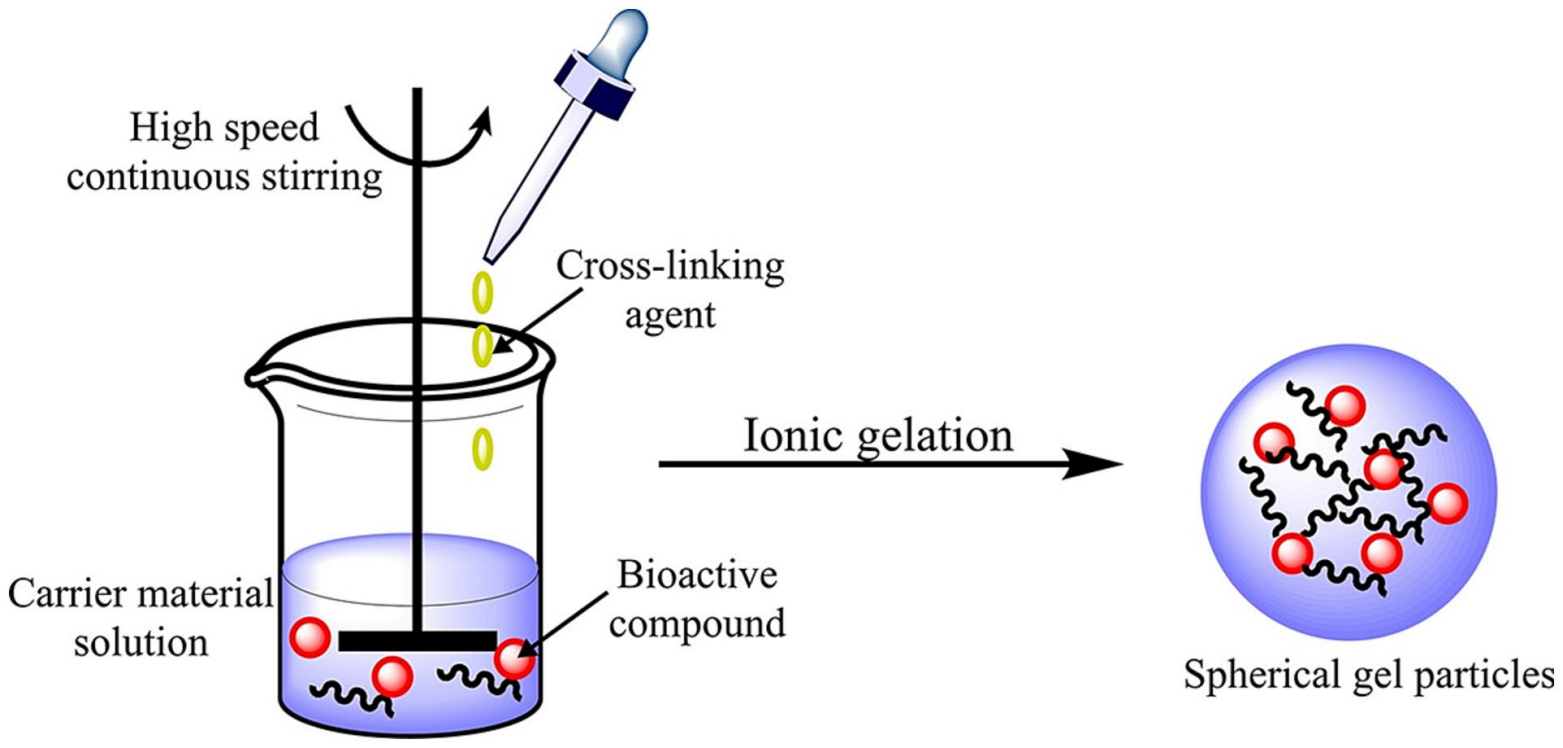
Schematic representation of the ionic gelation method. Adapted from (176), licensed under CC BY 4.0.

Ionotropic or ionic gelation is a widely used chemical encapsulation method that relies on electrostatic interactions between oppositely charged biopolymers and multivalent ions, leading to rapid formation of a three-dimensional cross-linked gel structure (124). In this technique, the bioactive compound is initially dissolved or uniformly dispersed within a polymer or hydrocolloid solution. The prepared mixture is then introduced into a solution containing cross-linking ions by dripping, extrusion, or atomization, while maintaining continuous agitation (125). Immediate gelation occurs when the polymer solution comes into contact with the ionic medium, producing spherical hydrogel particles that entrap the bioactive compounds (Figure 2). Natural polysaccharides, such as alginate, pectin, chitosan, and chitin, are frequently used as encapsulating matrices. At the same time, calcium ions (Ca^2+^) are most commonly used as cross-linking agents due to their strong interaction with carboxyl groups on these polymers (126). Depending on how the cross-linking ions migrate within the system, ionic gelation can occur through two different mechanisms: external and internal gelation. In external gelation, ions diffuse from the surrounding solution into polymer droplets, initiating gel formation. Conversely, internal gelation involves the gradual release of ions from within the polymeric matrix, typically through emulsified systems, which subsequently induces gel network formation (127). The versatility of ionic gelation has been demonstrated across a wide range of bioactive-rich plant matrices. Propolis, a resinous product rich in phenolic compounds and associated with multiple health benefits, illustrates the challenges commonly encountered in functional food applications, particularly poor water solubility and intense sensory attributes. Encapsulation using sodium alginate via ionic gelation has been proven effective in addressing these limitations by masking undesirable tastes and aromas while preserving antioxidant activity (128). Studies evaluating propolis extracts prepared in alcohol, honey, and glycerol have shown that increasing alginate concentration improves capsule uniformity and mechanical strength. Capsules produced with higher alginate levels effectively retained their structure during simulated gastric digestion, highlighting their potential for intestinal delivery. Among the solvent systems tested, honey-based propolis extracts yielded the most structurally robust and stable capsules, suggesting superior compatibility with alginate matrices and promising prospects for commercial applications (128).

Similarly, lemon balm (*Melissa officinalis* L.), a medicinal plant valued for its antioxidant and aromatic properties, has been successfully microencapsulated using ionic gelation. Extracts obtained from fresh plant material exhibited higher levels of bioactive compounds and were efficiently incorporated into alginate-based microcapsules. Spectroscopic analysis revealed minimal chemical alteration following encapsulation, while volatile profiling confirmed the effective retention of key terpenes and terpenoids, such as citral and caryophyllene. These findings underscore the suitability of ionic gelation for preserving both functional and sensory attributes of aromatic plant extracts (129). The encapsulation of fruit-derived bioactives has also benefited from ionic gelation strategies. Pitanga (*Eugenia uniflora* L.), a phenolic-rich tropical fruit with strong antioxidant capacity and attractive pigmentation, has been encapsulated using ionic gelation alone or in combination with drying techniques such as fluidized bed drying (130). Optimized conditions resulted in microparticles with enhanced stability of active compounds, supporting the use of encapsulated pitanga extracts as natural colorants or functional ingredients in food systems.

Advanced formulation approaches have further expanded the applicability of ionic gelation. For instance, calcium alginate–pectin beads incorporating papaya leaf extract have been developed and optimized using response surface methodology. Process variables, such as alginate concentration, calcium chloride concentration, and extrusion flow rate, were found to affect encapsulation efficiency and bead morphology significantly. Optimized beads demonstrated favorable storage stability and controlled release of polyphenols, reinforcing the relevance of such systems for functional food delivery (131). *Moringa oleifera*, a plant widely recognized for its high phenolic and antioxidant content, has been extensively studied in both leaf and seed extract forms. Hydroalcoholic seed extracts encapsulated via alginate-based ionic gelation exhibited superior color stability, moisture retention, and preservation of antioxidant activity compared to non-encapsulated extracts during refrigerated storage (132). Optimized formulations retained more than 85% of their antioxidant activity over 4 weeks.

The incorporation of multilayer coatings has further improved the functional performance of ionic gelation systems. The encapsulation of phenolic-rich grape pomace extracts using alginate alone or in combination with gelatin or chitosan demonstrated that secondary coatings significantly enhanced encapsulation efficiency and modified the bead morphology. These physicochemical changes directly influenced gastrointestinal behavior, leading to improved intestinal bioaccessibility of individual phenolic compounds. Alginate–gelatin systems exhibited the highest bioaccessibility indices, highlighting the importance of selecting tailored wall materials for targeted delivery (133, 134). Internal gelation, on the other hand, involves blending a mixture of a core, a charged biopolymer, and an anionic salt solution. To form small encapsulates, a prepared emulsion is added with continuous blending. Outer and internal structures of dispersed droplets or encapsulates are hardened by an anionic salt solution. A core material is entrapped and protected by the gel network (135). Adequate concentrations and a high degree of complex formation between biopolymers, as well as between biopolymers and anionic salts, result in complexes with a compact structure. In contrast, inadequate concentrations and a lower degree of complex formation or cross-linkage would result in a shrunken, rough, or porous structure, which would adversely affect the encapsulation efficiency (136).

### 3.2 Electrospinning

**Figure 3:**
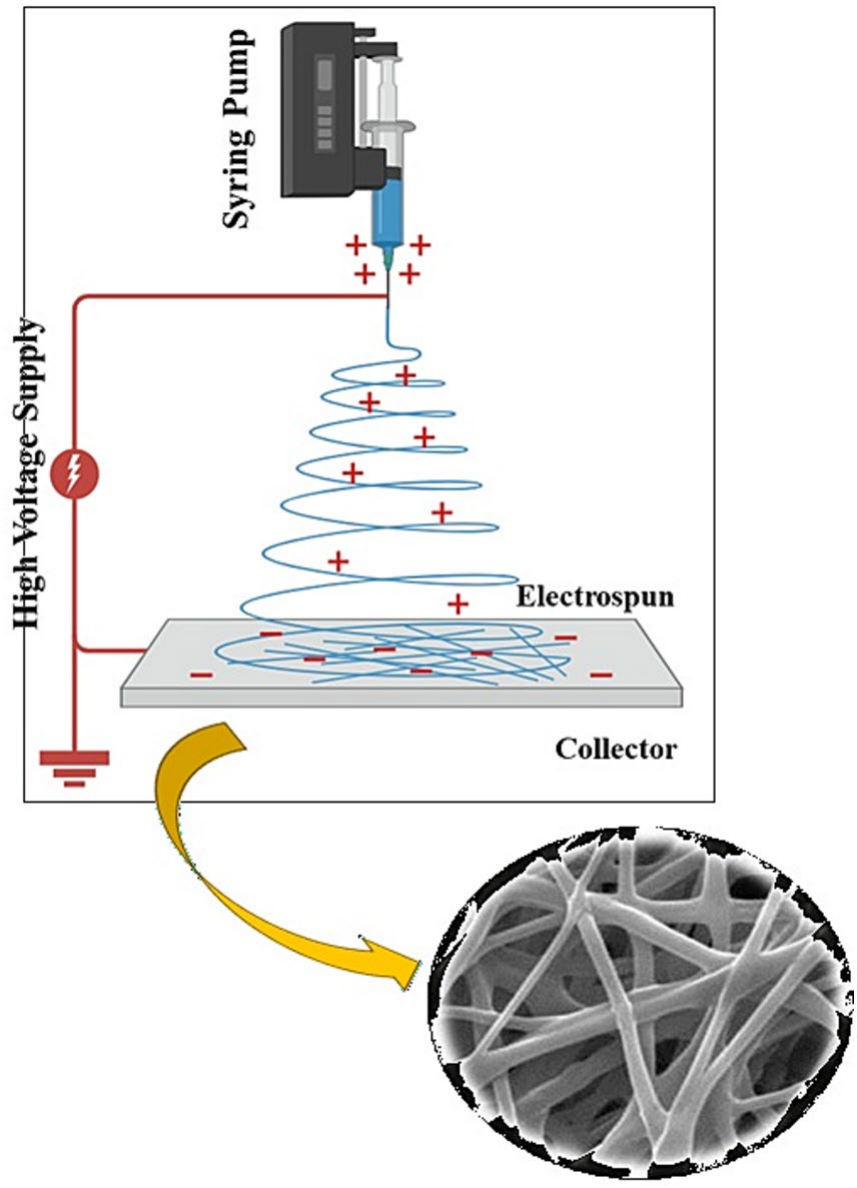
Electrospinning methods of encapsulation of bioactive compounds (137). Adapted from (168), licensed under CC BY 4.0.

Electrospinning (Figure 3) has emerged as a promising physical method for encapsulating and stabilizing sensitive bioactive compounds, owing to its ability to produce ultrafine polymeric nanofibers with high surface area, tunable morphology, and controlled release characteristics (137). Recent research has increasingly focused on polymeric nanofiber systems to enhance the stability, bioavailability, and controlled delivery of bioactive compounds for applications in functional foods, nutraceuticals, and pharmaceutical formulations. One notable application involves the valorization of agro-industrial by-products such as grape pomace, a major residue of red wine production that is rich in anthocyanins but highly susceptible to degradation. To address this limitation, grape pomace extract was encapsulated within electrospun ultrafine zein fibers at concentrations of 5, 10, and 15% (w/w) (138). The encapsulated extract demonstrated significant antioxidant activity, as evidenced by scavenging hydroxyl and nitric oxide radicals. Furthermore, the fibers exhibited antihyperglycemic potential by inhibiting carbohydrate-hydrolyzing enzymes, namely *α*-amylase and α-glucosidase, and anti-inflammatory activity by inhibiting thermal protein denaturation. The results highlight the potential of electrospun zein fibers as protective carriers for anthocyanin-rich extracts, facilitating their incorporation into functional foods and natural therapeutic formulations (138). Similarly, electrospinning has been explored for encapsulating plant extracts with antioxidant properties. In one study, gelatin (GL) and octenyl succinic anhydride-modified starch (OS-ST) were used to fabricate electrospun nanofibers incorporating 5% (v/v) sage extract (SE). Polymer solutions with GL/OS-ST ratios of 100/0, 90/10, 70/30, 60/40, and 50/50 (w/w) were electrospun to evaluate the influence of polymer composition on nanofiber characteristics (139). Increasing OS-ST content reduced solution viscosity and resulted in smaller fiber diameters, reaching approximately 231.62 nm. Antioxidant activity assays demonstrated sustained release of SE over 48 h, achieving radical scavenging activity of 72.3% ± 3%. Among the formulations, the GL/OS-ST ratio of 70:30 produced uniform nanofibers with the highest encapsulation efficiency of 87.5% ± 2.1%, highlighting their suitability as bioactive delivery systems (139). Electrospinning has also been effectively applied to encapsulate lipid-based bioactive compounds such as omega-3 fatty acids. In this approach, whey protein isolate (WPI) and polyvinyl alcohol (PVA) blends were used to fabricate nanofibers for the encapsulation of omega-3 fatty acids. Various WPI/PVA ratios (100:0, 90:10, 80:20, 70:30, 60:40, and 50:50 v/v) were evaluated based on surface tension, viscosity, and conductivity. Scanning electron microscopy (SEM) revealed that ratios of 90:10 and 80:20 produced uneven fibers with bead formation. In contrast, the 70:30 ratio yielded uniform, bead-free nanofibers with an average diameter of 262.7 ± 49.5 nm (140).

In addition to direct encapsulation approaches, emulsion electrospinning has been used to incorporate hydrophobic compounds, such as carotenoids, into nanofiber matrices. İnan-Çınkır et al. (141) encapsulated carotenoid microemulsions within zein nanofibers using response surface methodology to optimize electrospinning parameters. The optimal conditions were identified as 23 kV, 1.7 mL/h, and 12.75 cm needle-to-collector distance. The encapsulation efficiency and yield were 77.78 and 41.76%, respectively, and the zeta potential was −29.73 mV, indicating stable nanofiber systems. The incorporation of the microemulsion influenced fiber morphology and diameter and reduced the diffusion coefficient within the zein matrix. When incorporated into model food systems, zein nanofibers enhanced carotenoid stability during storage compared with conventional carriers such as olive oil, milk, and water, although slight color changes were observed (141). These studies demonstrate that electrospinning provides an efficient platform for encapsulating diverse bioactive compounds, including polyphenols, plant extracts, lipids, and carotenoids, while enhancing their stability, bioactivity, and controlled release. The ability to tailor polymer composition, electrospinning parameters, and fiber morphology further enables the design of advanced nanofiber-based delivery systems for functional food and nutraceutical applications.

### 3.3 Complex coacervation techniques

**Figure 4:**
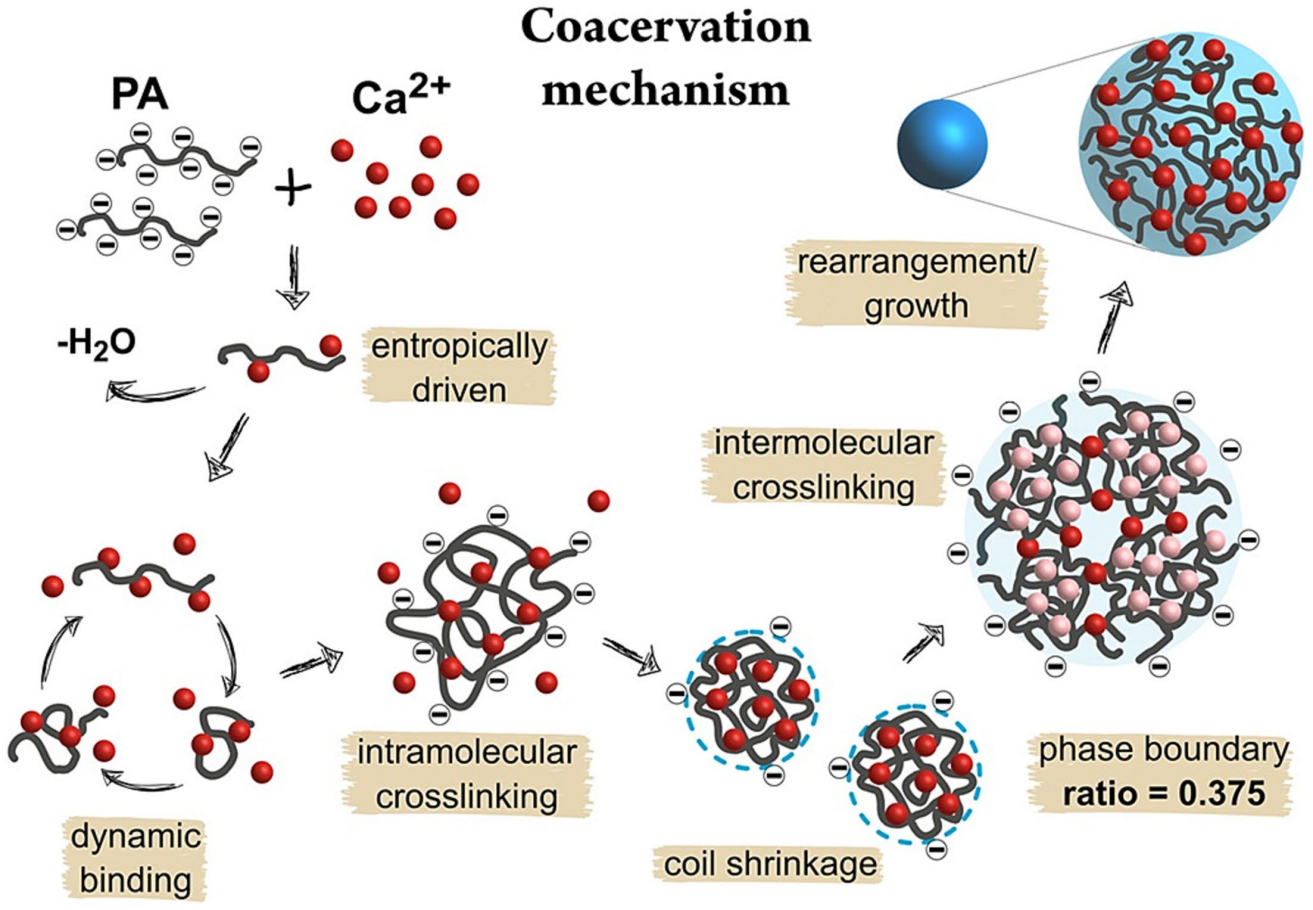
Schematic representation of complete coacervation mechanism starting from the initial calcium complexation, including the dynamic intra- and intermolecular binding behavior, finally resulting in reaching the phase boundary’s locus forming the coacervate electrostatically stabilized coacervate droplets. Reproduced from (177), licensed under CC BY 4.0.

Complex coacervation has emerged as an effective physicochemical method for encapsulating bioactive compounds, particularly to improve their stability, controlled release, and bioavailability in functional food and nutraceutical applications (142). This method relies on electrostatic interactions between oppositely charged biopolymers, typically proteins and polysaccharides, which form a coacervate phase capable of entrapping sensitive bioactive compounds (Figure 4). Several recent studies have demonstrated the versatility of complex coacervation for encapsulating plant extracts, essential oils, and other bioactive ingredients while preserving their functional properties. Pongpairoj et al. (143) valorise tender aromatic coconut mesocarp (TCM), an underutilized byproduct of coconut processing, as a natural source of phenolic antioxidants. Although TCM extract possesses strong antioxidant activity, its low stability under gastrointestinal conditions restricts its practical applications. Microcapsules were prepared using soy protein isolate (SPI) combined with gum arabic (GA), inulin (IN), or sodium alginate (SA), with the core concentration varied. Among the tested formulations, the SPI–inulin system at 0.1% core concentration exhibited the highest encapsulation efficiency of 98.74%, along with improved antioxidant recovery following *in vitro* digestion. The resulting microcapsules also displayed favorable physicochemical properties, including small particle size and a positive surface charge, which contributed to their structural stability (143). Similarly, complex coacervation has been successfully applied to encapsulate propolis extract using whey proteins and gum arabic as wall materials. In this system, the microcapsules were produced through complex coacervation followed by oven drying. The process achieved an encapsulation efficiency of 80.3% and a yield of 74.3%, producing microparticles with an average diameter of 3.14 μm. Importantly, the encapsulation process effectively preserved the antioxidant activity of propolis extract (144).

Complex coacervation has also been investigated as an effective strategy for the co-encapsulation of multiple bioactive compounds, enabling synergistic delivery and improved functional performance. In a recent study, saffron petal extract (SPE) and *Stachys schtschegleevii* extract (SSE) were simultaneously encapsulated using a chitosan–gum arabic biopolymer system through complex coacervation, followed by spray-drying to obtain stable microcapsules (145). Prior to spray drying, the formed coacervates were blended with maltodextrin, basil seed gum, and graphene oxide (GO) at concentrations of 0.1–0.2%. The resulting SPE and SSE coacervates exhibited average particle sizes of 385.32 nm and 579.06 nm, with corresponding polydispersity indices of 0.30 and 0.32, respectively. Morphological analysis revealed that the final co-encapsulated powders possessed spherical particles with slightly wrinkled surfaces, while surface-associated nanoparticles ranging from 141 to 291 nm were also observed. Factors, including the encapsulation sequence, graphene oxide concentration, and the nature of the core material, strongly influenced the release behavior of anthocyanins from SPE and D-germacrene from SSE. Importantly, the co-encapsulation system improved the bioavailability of both compounds by enabling their simultaneous delivery, resulting in an average intestinal release of approximately 88% (145). Another application of complex coacervation involves encapsulating essential oils to improve oxidative stability and control release. For instance, *Citrus limon* essential oil (CEO), known for its characteristic aroma and health benefits, has been encapsulated using gelatin and carrageenan as oppositely charged biopolymers. The highest encapsulation performance was achieved with 1.4 g gelatin, 0.2 g carrageenan, and 0.6 mL CEO, yielding 93% encapsulation and 95% efficiency. Although the gelatin–carrageenan system did not completely prevent the early release of monoterpenes, it allowed a delayed release of less volatile sesquiterpenes (146). These studies demonstrate that complex coacervation is a versatile and efficient encapsulation technique capable of protecting diverse bioactive compounds, improving their physicochemical stability, and enabling controlled release under gastrointestinal conditions. By tailoring the combination of proteins, polysaccharides, and processing conditions, this approach can be optimized for a wide range of functional food and nutraceutical applications.

### 3.4 Liposome-assisted encapsulation

**Figure 5:**
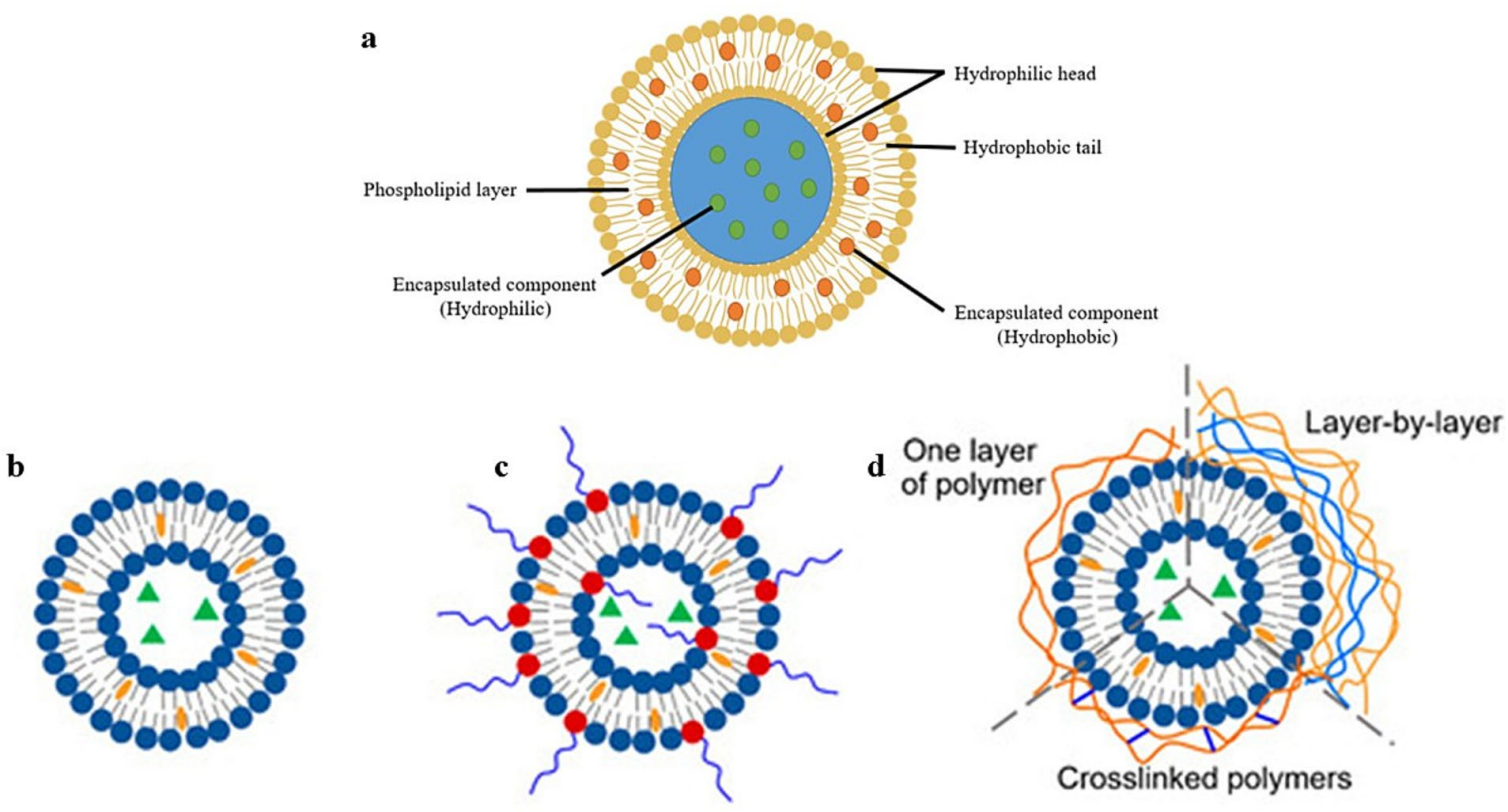
Schematic representation of **(a)** liposome showing the phospholipid bilayer with hydrophilic heads, hydrophobic tails, and the localization of encapsulated hydrophilic and hydrophobic c ompounds (178), **(b)** conventional liposome (179), **(c)** polymer-“grafted” liposome (179), and **(d)** polymer-coated liposome, where liposomes are coated by a layer of adsorbed polymer, by the layer-by-layer (LbL) assembly, or by crosslinked polymers (polymer-“caged”). Adapted from (178) and (179), licensed under CC BY 4.0.

Liposome delivery systems, a physicochemical method of encapsulation (Figure 5), have been widely investigated for the stabilization and controlled delivery of bioactive compounds in functional foods and nutraceutical formulations. These approaches improve the solubility, protection, and bioavailability of poorly water-soluble phytochemicals while enhancing their stability during processing, storage, and gastrointestinal digestion. Propolis, a natural bee-derived product, is well recognized for its strong antioxidant, antimicrobial, and antifungal activities. However, the poor aqueous solubility of several phenolic constituents limits their effective utilization in food systems. To address this challenge, Ozkan, Ugur, and Capanoglu (147) developed an ultrasonication-assisted liposomal encapsulation approach to enhance the incorporation of propolis phenolic compounds. Propolis extract concentrations ranging from 0.05 to 0.5% were evaluated to determine the optimal loading capacity. The EE values ranged from 52.76 to 76.12%, with secondary liposomes containing 0.05% propolis extract exhibiting the highest EE. Additionally, incorporating propolis-loaded liposomes into apple juice significantly improved the stability and preservation of bioactive compounds during digestion, highlighting the potential of liposomal carriers for functional food applications. Similarly, the incorporation of phytosterols into liposomal systems has been explored to improve the physicochemical stability and functional performance of encapsulated plant extracts. Tripathy and Srivastav (107) studied the effect of β-sitosterol (βS) on the structural properties, stability, and release behavior of liposomes containing *Centella asiatica* leaf extract (CALE). Among the formulations tested, the highest encapsulation efficiency (74.789 ± 0.811%) was achieved with a soy lecithin (SL) to β-sitosterol (βS) ratio of 7:3 (LP-βS (C3)). All liposomal formulations exhibited particle sizes below 700 nm and maintained a retention rate exceeding 50% after 28 days of storage, indicating satisfactory storage stability. Moreover, simulated release studies indicated that liposomal encapsulation enhanced the bioavailability of CALE polyphenols (107). Another example involves the encapsulation of mangiferin, a natural polyphenolic compound known for its diverse physiological activities. Despite its therapeutic potential, mangiferin exhibits limited oral bioavailability due to its poor solubility and instability in the gastrointestinal environment. To overcome these limitations, mangiferin-loaded liposomes were prepared using the ethanol injection method, and the optimal formulation parameters were determined through single-factor and orthogonal experimental designs based on entrapment efficiency (148). The prepared liposomes were subsequently modified using a layer-by-layer coating strategy with whey protein and *κ*-carrageenan, resulting in three different formulations: mangiferin liposomes (ML), whey protein-coated mangiferin liposomes (W-ML), and whey protein–κ-carrageenan modified mangiferin liposomes (W/C-ML). Comparative analysis revealed that the double-layer modified liposomes (W/C-ML) achieved the highest entrapment efficiency (70.63% ± 0.86%) and demonstrated improved physical stability compared with the other formulations. *In vitro* digestion studies further showed that the W/C-ML formulation exhibited the highest release and cumulative release rates in simulated intestinal fluid, indicating enhanced bioavailability of mangiferin (148). These studies demonstrate that lipid-based encapsulation strategies, including multilayer liposomes and sterol-stabilized systems, can significantly improve the stability, controlled release, and bioavailability of plant-derived bioactive compounds. Such approaches provide promising opportunities for the development of advanced delivery systems in food, pharmaceutical, and nutraceutical applications.

### 3.5 Fluidized bed-assisted encapsulation

Fluidized bed coating, a physical method of encapsulation, has emerged as an effective post-encapsulation strategy to enhance the stability, controlled release, and functional performance of bioactive compounds. It is particularly valuable when combined with primary encapsulation techniques such as spray drying or spray chilling, enabling the formation of multilayered delivery systems with improved barrier properties (149). The development of a dual-layer microencapsulation system for grape skin extract, a phenolic-rich by-product of the food industry. Initially, spray chilling with palm oil was employed for primary encapsulation, followed by a secondary zein coating. Although the primary encapsulation step defines the core structure, the secondary layer, achieved through coating methods such as fluidized-bed processing, plays a critical role in modulating release and enhancing protection (150). The resulting microcapsules demonstrated low moisture content (5.50 ± 0.08%), reduced water activity (0.38 ± 0.02), and high encapsulation efficiency (98.98 ± 1.11%). In vitro gastrointestinal studies revealed minimal resveratrol release in the oral and gastric phases, with a significant increase in the intestinal phase due to enzymatic lipid degradation (150). These findings highlight that secondary coatings, including those applied via fluidized-bed systems, significantly improve oxidative stability and enable targeted intestinal delivery.

Fluidized bed coating is also widely applied to enhance spray-dried microparticles. For instance, linseed oil microcapsules produced using maltodextrin and whey protein were further coated with hydroxypropyl methylcellulose (HPMC) in a fluidized bed system. Process optimization using a central composite rotatable design showed that a coating solution flow rate (Q) of 1.87 mL/min and fluidizing air temperature (T) of 71 °C yielded optimal properties, including a surface oil content of 10.4% and water activity of 0.484 (151). Functionally, the coated microparticles exhibited significantly reduced lipolysis compared to uncoated ones, with maximum free fatty acid release of 22.38% versus 48.14%, respectively.

Beyond encapsulation, fluidized bed technology is also applied in agglomeration processes to improve the incorporation and stability of bioactive-rich ingredients in food systems. For example, *Spirulina platensis* powder was agglomerated with 30% maltodextrin in a fluidized bed prior to incorporation into yogurt at 0.5–2.0% (w/v). Fermentation was conducted at 42 °C for 5 h, followed by storage at 4 °C for 28 days (152). The resulting yogurts met regulatory standards, with total acidity ranging from 0.6–1.5%, pH between 3.5–4.6, and viable lactic bacteria counts of at least 10^7^ CFU·g^−1^. Nutritional enhancement was evident, with protein content increasing to 4.2–5.6% and ash content to 1.3–1.8%. Bioactive enrichment included phenolic compounds (2.98–14.96 mg·100 g^−1^), phycocyanin (2.19–3.65 mg·100 g^−1^), *β*-carotene (4.73–6.37 mg·100 g^−1^), and chlorophyll a (12.39–13.77 mg·100 g^−1^) (152). Notably, agglomeration improved the retention and stability of these bioactives during fermentation and storage, demonstrating the effectiveness of fluidized bed processing in preserving functional compounds. Fluidized bed methods, whether used for coating or agglomeration, significantly enhance the functional performance of encapsulated bioactive compounds. They provide enhanced oxidative protection, controlled release, and improved integration into food matrices, making them highly suitable for the development of next-generation functional foods and nutraceuticals.

### 3.6 High pressure homogenization

**Figure 6:**
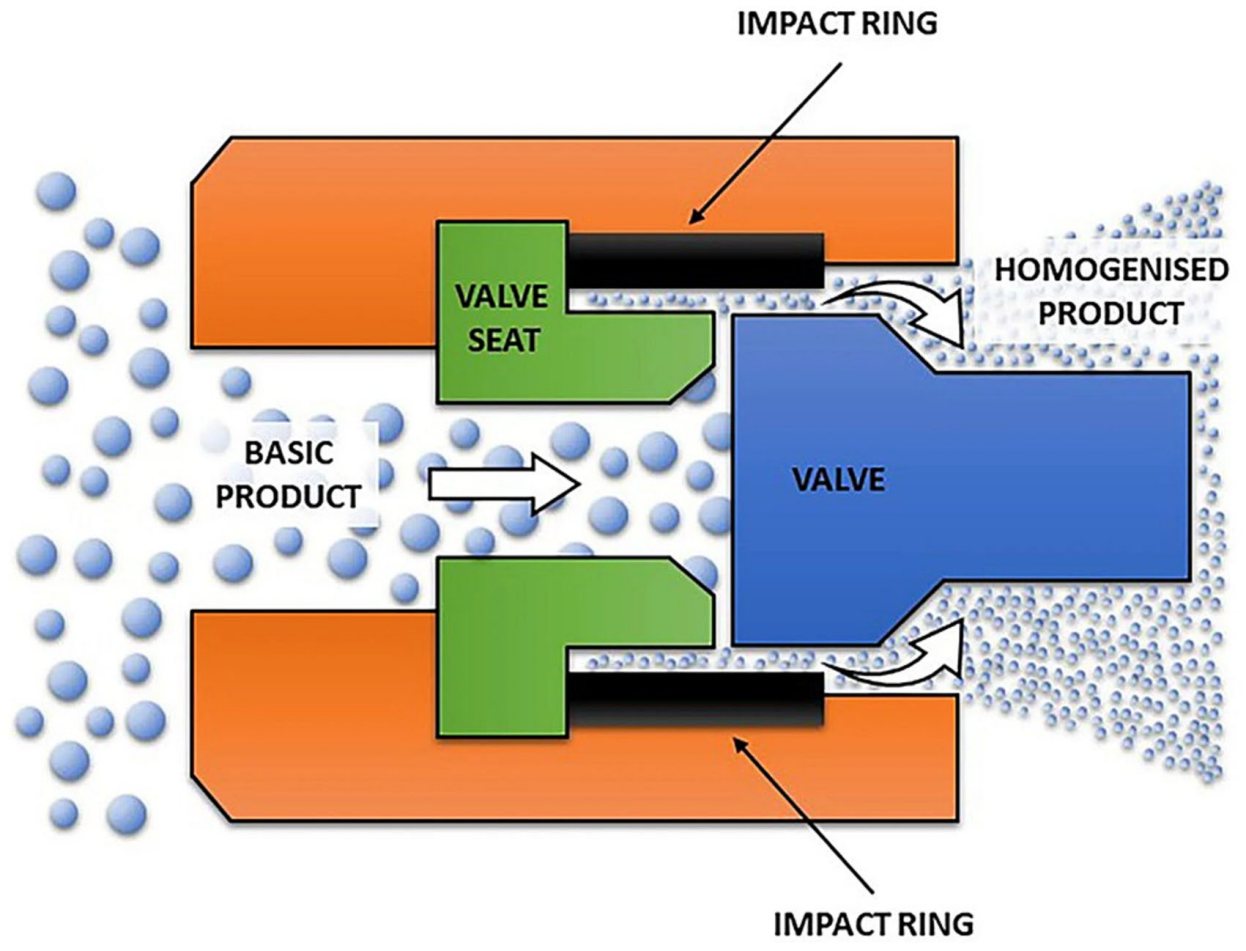
Schematic representation of high-pressure homogenization technology. Adapted from (180), licensed under CC BY 4.0.

High-pressure homogenization (HPH) is a physicochemical method widely used to improve the stability of sensitive bioactive compounds, such as astaxanthin, which is highly susceptible to degradation by light, heat, and oxidative conditions (Figure 6). To address these limitations, astaxanthin was formulated into a nanoemulsion (ANE) and further coated with chitosan (CS) to form CS-ANE systems using HPH. The effects of processing parameters, including homogenization pressures of 150 and 180 MPa, 1–4 homogenization cycles, and varying CS concentrations (0% (CS0-ANE), 0.55% (CS1-ANE), 0.75% (CS2-ANE), and 0.95% (CS3-ANE)), were systematically evaluated (153). Increasing pressure and the number of cycles reduced droplet size, while CS incorporation increased zeta potential, indicating improved colloidal stability. Encapsulation efficiency (EE) increased markedly from 59.61% in CS0-ANE to 76% (CS1-ANE), 87.04% (CS2-ANE), and 90.28% (CS3-ANE). Notably, CS2-ANE and CS3-ANE maintained thermal stability at 90 °C for 30 min and demonstrated superior retention of antioxidant activity compared to formulations with lower CS content. In addition to DPPH and FRAP assays, performance in a real food system showed that CS2-ANE was most effective in suppressing lipid oxidation in safflower oil stored at 45 °C (153). Similarly, HPH has been used to encapsulate natural food colorants in liposomal systems to improve their physicochemical and functional properties. In this approach, liposomes containing anthocyanins from freeze-dried raspberry powder (R), copper chlorophyllin complexes (C), and β-carotene (B) were prepared using sunflower lecithin and carboxymethylcellulose sodium salt as stabilizing agents. The resulting dispersions exhibited median particle sizes of approximately 200 nm for R and C formulations, whereas B-containing systems showed a wider size distribution ranging from 165 to 405 nm (154). Rheological analysis indicated that flow behavior varied with applied shear, suggesting structural rearrangements within the dispersions. The systems displayed a translucent appearance, with high lightness values and characteristic hue angles (h*) for each encapsulated dye. Zeta potential values around −30 mV confirmed good electrostatic stability. Encapsulation efficiency (EE) differed notably among the dyes, with anthocyanins achieving the highest EE (36.17–84.61%), while chlorophyll derivatives showed lower efficiencies (1.82–16.03%) (154).

In another application, HPH combined with a pH-shift technique was used to enhance the stability and bioaccessibility of pterostilbene (PTS) through complexation with hydroxypropyl-β-cyclodextrin (HP-β-CD). The encapsulation process, conducted at pressures of 50, 100, and 150 MPa with three treatment variations, achieved a maximum EE of 94.34% and a loading capacity of 14.82% at 150 MPa (155). The encapsulated PTS demonstrated improved stability, retaining 82.22% at 4 °C over 28 days and 76% after 120 min of UVB exposure, compared to 56.03 and 21% for free PTS. Antioxidant activity was also enhanced, with a DPPH scavenging rate of 44.95% at 0.02 mg/mL, nearly double that of the non-encapsulated compound. Release kinetics followed the Higuchi model, and *in vitro* digestion studies showed reduced release in gastric (32.24% vs. 49.88%) and intestinal (63.98% vs. 91.01%) phases. Furthermore, no cytotoxicity was observed in human epidermal keratinocytes, indicating good biocompatibility (155). These findings highlight the effectiveness of HPH-assisted encapsulation strategies in improving the functional stability and delivery of pterostilbene for potential applications in food, pharmaceutical, and cosmetic systems.

### 3.7 Extrusion-spheronization based encapsulation

Extrusion–spheronization (ES) is an established pelletization technique traditionally used in pharmaceutical formulation. Still, its potential for encapsulating bioactive compounds for food and nutraceutical applications has increasingly attracted attention due to its ability to produce spherical, dense, uniform pellets with controlled release characteristics, high encapsulation efficiency, and improved stability (156). The process typically involves wet massing of bioactive-loaded excipients, extrusion through a die, and spheronization to form spherical pellets, which can be further coated or modified for targeted release. These features make ES particularly attractive for functional food ingredients, nutraceutical delivery systems, and stabilization of sensitive food bioactives.

One of the promising applications of ES in food-related bioactive delivery is the encapsulation of essential oil compounds. Carvacrol, a major phenolic constituent of *Thymus vulgaris* essential oil with recognized antioxidant, antimicrobial, and therapeutic activities, has been successfully encapsulated using a combined liquisolid-extrusion-spheronization approach (157). In this system, liquid carvacrol was converted into a solid-state formulation using carriers such as polyvinylpyrrolidone (PVP), followed by pellet formation through ES. The resulting pellets exhibited desirable physicochemical properties, improved stability, and controlled release behavior (157). From a food application perspective, such encapsulated carvacrol systems offer significant promise as functional ingredients or natural preservative delivery vehicles in food matrices, where volatility, oxidation, and uncontrolled release of essential oil components are major limitations. The ability of ES to transform unstable lipophilic bioactives into stable, free-flowing pelletized systems can facilitate incorporation into powdered foods, dietary supplements, and controlled flavor or antimicrobial release applications.

Extrusion-spheronization has also demonstrated substantial potential for probiotic encapsulation, particularly in developing targeted gastrointestinal delivery systems for functional foods. Freeze-dried *Lactobacillus reuteri* has been incorporated into ES-produced pellets, followed by pH-responsive fluid-bed coating to achieve delayed release in the ileum. This approach significantly improved probiotic protection against gastric conditions while maintaining viability until intestinal release (158). Such delivery systems have important implications for next-generation synbiotic and probiotic food formulations, including fortified beverages, nutraceutical capsules, and functional food supplements, where survivability of probiotic cultures during processing, storage, and gastrointestinal transit remains a critical challenge. The dense, uniform pellet structure produced by ES, combined with coating compatibility, provides a robust strategy for the controlled delivery of sensitive microbial bioactives in food applications.

Beyond probiotics and essential oils, modified extrusion–spheronization has also been explored for the encapsulation of unstable or hygroscopic nutraceutical compounds. Choline bitartrate, an important nutrient associated with fetal brain development and cognitive health, has been successfully microencapsulated using hydrogenated soybean oil as a food-compatible encapsulating matrix through modified ES. The resulting pellets exhibited excellent encapsulation efficiency, storage stability, and controlled release, overcoming issues such as moisture sensitivity, discoloration, and odor generation (159). Such findings highlight the relevance of ES in fortification technologies, where micronutrients and labile bioactives can be incorporated into stable pelletized delivery systems for fortified foods, dietary supplements, or functional ingredient premixes. Although some ES studies have focused on pharmaceutical actives such as atenolol using lipidic and polymeric matrices, they provide important formulation insights transferable to food bioactive encapsulation. The successful use of hydrophobic waxes, such as carnauba wax and glyceryl monostearate, combined with process modifications, such as sintering and compaction, to modulate release, demonstrates the adaptability of ES for designing sustained or site-specific release systems using food-grade excipients (160). Such strategies could be translated into controlled-release delivery of plant polyphenols, antioxidants, omega-3 oils, or other nutraceuticals in food systems.

Extrusion–spheronization offers several advantages for food bioactive encapsulation, including the use of food-compatible carriers, high loading capacity, improved oxidative and storage stability, controlled or targeted release, and compatibility with downstream coating technologies. Despite these advantages, its application in food systems remains relatively underexplored compared with spray drying, ionic gelation, or fluidized-bed encapsulation. Existing studies have primarily focused on probiotics, essential oil constituents, and select nutraceuticals, suggesting considerable opportunity to expand ES to encapsulate plant-derived polyphenols, agro-industrial bioactives, and functional ingredients for innovative food product development. This emerging area presents significant scope for developing structured pelletized delivery systems with improved functionality and stability for advanced food applications.

## 4 Enhanced delivery and controlled release of bioactive compounds

### 4.1 Protection, stability, and bioaccessibility

Bioactive compounds such as polyphenols, carotenoids, vitamins, essential oils, and bioactive peptides have attracted considerable interest in functional foods and nutraceutical formulations because of their antioxidant, anti-inflammatory, antimicrobial, and disease-preventive properties (161). However, their practical application is often limited by poor physicochemical stability, low solubility, and rapid degradation during processing, storage, and gastrointestinal digestion (9). These compounds are highly susceptible to environmental stressors such as heat, light, oxygen, moisture, and pH fluctuations, which can significantly reduce their biological activity and shelf life (7, 162). Encapsulation technologies have emerged as an effective strategy to protect sensitive bioactive compounds from degradation and enhance their delivery efficiency. Encapsulation involves entrapping bioactive molecules within a protective carrier or wall material that shields them from environmental stressors and regulates their release under specific physiological conditions. This approach not only improves the stability of bioactive compounds but also enhances their bioavailability and functional performance in food systems (11, 104). One of the primary advantages of encapsulation is the enhancement of chemical and physical stability during food processing and storage. For instance, citrus-derived bioactive compounds, such as flavonoids, carotenoids, and vitamin C, are highly sensitive to oxidative degradation and environmental stresses, which significantly limit their use in functional foods (163). Encapsulation techniques such as spray drying, freeze drying, and nanoencapsulation provide protective matrices that prevent degradation and maintain their antioxidant activity and nutritional value (7).

Similarly, bioactive phytochemicals obtained from agro-industrial by-products, such as pomegranate peel and seed extracts, contain phenolic compounds, flavonoids, and ellagitannins with strong antioxidant and antimicrobial properties (164). Despite their health benefits, these compounds are unstable under environmental conditions, including light, oxygen exposure, and temperature variations. Microencapsulation has been widely used to stabilize these compounds, enabling their incorporation into food products while preserving biological activity and extending shelf life (165). Encapsulation systems also play a crucial role in improving the bioaccessibility of bioactive compounds. Bioaccessibility refers to the fraction of a compound released from the food matrix during digestion and made available for intestinal absorption (8). Many bioactive compounds exhibit low bioaccessibility due to poor water solubility and limited interaction with digestive enzymes (3). Delivery systems such as lipid-based nanoparticles, nanoemulsions, and structured lipid carriers can significantly enhance the dispersibility and solubility of hydrophobic compounds, thereby increasing their bioaccessibility during gastrointestinal digestion (6).

Nanostructured lipid carriers (NLCs) prepared from edible lipids such as beeswax and flaxseed oil have demonstrated high encapsulation efficiency and improved stability of hydrophobic bioactives. Ma et al. (166) encapsulated *β*-sitosterol in beeswax–flaxseed oil NLCs, achieving an encapsulation efficiency of approximately 92% and maintaining physical stability for 40 days under various pH and temperature conditions. Moreover, *in vitro* digestion experiments revealed that these carriers significantly increased the bioaccessibility of β-sitosterol to 43.9%, compared with only 12.1% for the non-encapsulated compound, demonstrating the effectiveness of lipid-based delivery systems for improving gastrointestinal stability and controlled release (166). Colloidal delivery systems, such as emulsions and Pickering emulsions, have also been widely explored to improve the stability and bioaccessibility of lipophilic bioactive compounds (12). Pickering emulsions stabilized by solid colloidal particles form a robust interfacial barrier that prevents droplet coalescence, oxidation, and degradation of encapsulated bioactive compounds. These systems have shown enhanced resistance to physicochemical destabilization and improved release of bioactive compounds during gastrointestinal digestion, making them promising carriers for functional food applications (19).

In addition to lipid-based carriers, natural polymers such as proteins, polysaccharides, and gums are commonly used as encapsulating materials due to their biocompatibility, biodegradability, and Generally Recognized as Safe (GRAS) status (5). These polymers can form stable networks that can entrap bioactive compounds, protect them from degradation, and enable controlled release. Polysaccharide-based carriers often form porous matrices that retain bioactive compounds efficiently, whereas protein and lipid carriers interact with hydrophobic compounds through specific molecular interactions, further enhancing stability and encapsulation efficiency (4, 167). Recent developments in hybrid nanoencapsulation systems have further improved the protective performance of encapsulation technologies. These systems integrate natural polymers with synthetic nanomaterials to combine the biocompatibility of natural carriers with the structural precision and tunable properties of engineered materials (20). Such hybrid systems have demonstrated improved encapsulation efficiency, enhanced resistance to environmental stressors, and better control over the release behavior of encapsulated bioactive compounds (20). The development of advanced encapsulation and delivery systems has significantly improved the protection, stability, and bioaccessibility of bioactive compounds. By protecting sensitive molecules from environmental degradation, improving their solubility and gastrointestinal stability, and enabling controlled release during digestion, encapsulation technologies provide an effective strategy for enhancing the functional performance of bioactive compounds in food, nutraceutical, and pharmaceutical applications.

### 4.2 Targeted and stimuli-responsive delivery systems

The effectiveness of bioactive compounds in functional foods, nutraceuticals, and biomedical applications is often limited by poor stability, rapid degradation, and uncontrolled release during digestion or physiological processes. To overcome these limitations, targeted and stimuli-responsive delivery systems have been developed to enable controlled release of bioactive compounds at specific sites under defined environmental triggers (22). These advanced delivery systems can respond dynamically to physiological stimuli, including pH changes, temperature fluctuations, enzymatic activity, light irradiation, magnetic fields, and redox conditions, enabling site-specific delivery and improved therapeutic efficacy. Stimuli-responsive delivery systems are often referred to as “smart delivery systems” because they are engineered to respond selectively to endogenous or exogenous signals present in biological environments (24). These systems allow controlled and on-demand release of encapsulated compounds at the desired site of action while minimizing premature release and degradation during processing, storage, or gastrointestinal transit.

Among the various stimuli-responsive systems, pH-responsive delivery platforms have received significant attention due to the substantial pH variations encountered along the gastrointestinal tract and in various pathological environments. These systems are designed to remain stable under acidic gastric conditions and subsequently release their payload under neutral or slightly alkaline conditions in the intestine, enabling site-specific release of bioactive compounds (168). For instance, polymer-complexed liposomal nanocarriers, commonly referred to as capsosomes, have been developed as pH-responsive systems for oral delivery of biomolecules. These nanocarriers consist of liposomal compartments assembled with chitosan-coated solid lipid nanoparticles, enabling electrostatic interactions that stabilize the structure during gastric transit. Under simulated gastrointestinal conditions, the capsosomes remain stable in acidic environments but disassemble at neutral pH (approximately pH 7.0), releasing up to 87% of the encapsulated liposomes in the small intestine, thereby improving intestinal delivery and retention time (26). Similarly, polysaccharide-based nano-delivery systems have been extensively investigated for pH-responsive delivery of bioactive ingredients. Polysaccharides such as starch, pectin, chitosan, alginate, and xanthan gum possess functional groups that respond to environmental pH variations, enabling the development of nanoparticles, nanogels, nanoemulsions, and nanocapsules capable of targeted release in the intestine. These systems improve the water dispersibility, stability, and controlled release of bioactive ingredients while preventing premature degradation during processing and digestion (25).

Hydrogel-based systems are also widely explored as stimuli-responsive delivery platforms due to their ability to undergo structural changes in response to environmental triggers. Natural polymer hydrogels can respond to pH, ionic strength, or temperature variations, enabling controlled diffusion and release of encapsulated bioactive compounds (23). A recent example involves the development of *κ*-carrageenan and dialdehyde starch-based emulsion gels for the delivery of hydrophobic bioactive compounds such as curcumin. In this system, dialdehyde starch forms crosslinks with κ-carrageenan through hemiacetal reactions, creating a porous gel network capable of encapsulating hydrophobic compounds. The resulting emulsion gel exhibits improved structural integrity, high encapsulation efficiency, and sustained release under simulated intestinal conditions, demonstrating its potential as a green, biocompatible platform for the oral delivery of bioactive compounds (169).

Nanocarriers represent another important class of targeted delivery systems that can improve the bioavailability and therapeutic efficacy of bioactive compounds (21). Various nanocarriers, including nanoparticles, liposomes, niosomes, and polymeric nanostructures, have been developed to deliver bioactive molecules to specific tissues or cells. For example, multifunctional niosomal nanoparticles have been investigated for the co-delivery of curcumin and microRNA-34a for cancer therapy (170). These nanoscale vesicular carriers, formed from nonionic surfactants and cholesterol, exhibit high encapsulation efficiency and enhanced cellular uptake. *In vitro* and *in vivo* studies demonstrated that co-delivery of curcumin and miR-34a using cationic niosomal nanoparticles resulted in significantly greater tumor inhibition than free compounds or single-agent treatments. This enhanced therapeutic effect was attributed to improved cellular uptake, controlled release, and targeted accumulation of the nanocarriers in tumor tissues (170). Targeted and stimuli-responsive delivery systems represent a promising strategy for enhancing the stability, bioavailability, and controlled release of bioactive compounds. By integrating smart materials with advanced encapsulation technologies, these systems can significantly improve the functional performance of bioactive compounds and support the development of next-generation functional foods and nutraceutical products.

### 4.3 Role of particle size on the bioactive delivery

Particle size is a critical design parameter in encapsulation systems, significantly influencing the stability, release kinetics, and bioavailability of bioactive compounds (171). At the macroscale (>100 μm), encapsulation systems such as beads, capsules, and hydrogels primarily provide physical protection and structural integrity. These systems are particularly effective at shielding sensitive compounds from environmental stressors, such as oxygen, light, and moisture, during processing and storage (172). However, due to their relatively large size and low surface area-to-volume ratio, macro-scale particles often exhibit limited dissolution and slower release rates, thereby limiting the bioaccessibility of encapsulated compounds. For instance, alginate-based macrobeads encapsulating polyphenols have demonstrated strong protection during gastric conditions but delayed release in intestinal environments, limiting rapid absorption (131). Despite this limitation, macro-scale systems are advantageous for applications requiring prolonged release or targeted delivery in the colon.

Micro-scale particles (100 nm–100 μm) represent a balance between protection and bioavailability and are widely used in food and nutraceutical applications (173). Their increased surface area enhances dispersibility and interaction with digestive fluids, leading to improved release and absorption compared to macro-systems (174). Microencapsulation techniques such as spray drying and complex coacervation have been shown to improve the stability and controlled release of bioactive compounds. For example, Bispo et al. (144) reported that propolis microcapsules prepared using whey protein and gum arabic exhibited high encapsulation efficiency (80%) and preserved antioxidant activity while enabling gradual release during digestion. Similarly, microencapsulated phenolic extracts from pitanga demonstrated enhanced stability and improved bioaccessibility due to optimized particle size and matrix structure (130). These findings indicate that micro-scale systems are particularly suitable for functional food applications that require both protection and efficient release.

In contrast, nanoscale delivery systems (1–100 nm) offer superior performance in terms of bioavailability, cellular uptake, and targeted delivery. The extremely small particle size results in a high surface area, improved solubility, and enhanced interaction with biological membranes, facilitating efficient transport across intestinal barriers (175). Nanocarriers such as nanoemulsions, liposomes, and polymeric nanoparticles have demonstrated significant improvements in the delivery efficiency of poorly soluble bioactive compounds. For instance, Fan et al. (88) developed soy protein–fucoidan nanoparticles for curcumin delivery with improved dispersion stability and bioavailability. Similarly, nanoliposomes loaded with plant extracts have shown enhanced gastrointestinal stability and increased intestinal absorption due to their ability to protect bioactives and promote cellular uptake (148). However, nanoscale systems also pose challenges related to aggregation, stability, scalability, and regulatory concerns that must be carefully addressed for practical applications. Tailoring particle size across macro, micro, and nanoscales provides a strategic approach to optimize encapsulation performance, enabling the design of efficient delivery systems for diverse bioactive compounds.

## 5 Challenges, limitations, and future perspectives

Despite the significant progress achieved in encapsulation technologies, several challenges remain that limit their large-scale implementation in food, nutraceutical, and pharmaceutical applications. One of the primary challenges is the stability of encapsulating systems during food processing and storage. Many encapsulation systems may undergo structural changes when exposed to high temperatures, mechanical stress, oxidation, or pH variations, which can compromise the protective capacity of the carrier matrix and lead to premature release of bioactive compounds. Another important limitation involves the scalability and economic feasibility of advanced encapsulation technologies. Techniques such as electrospinning and multilayer liposomal systems often require specialized equipment and complex processing conditions, which may increase production costs and hinder industrial adoption. Furthermore, achieving consistent particle size distribution, high encapsulation efficiency, and reproducible release behavior remains challenging at large scale. Safety and regulatory considerations also represent important challenges, particularly for nano-based delivery systems. Although many encapsulating materials, such as proteins, polysaccharides, and lipids, are Generally Recognized as Safe (GRAS), the long-term safety and regulatory approval of certain nanostructured carriers require further evaluation. Additionally, potential interactions between encapsulated bioactive compounds and food matrices must be carefully studied to ensure product stability and consumer safety.

Future research should focus on developing sustainable, cost-effective encapsulation strategies using biodegradable, food-grade materials. The integration of nanotechnology, biomaterials engineering, and computational modeling could facilitate the design of multifunctional delivery systems with enhanced encapsulation efficiency and controlled release. Furthermore, the development of stimuli-responsive and targeted delivery systems that respond to gastrointestinal conditions, enzymatic activity, or environmental triggers represents a promising approach to enhancing bioactive compound bioavailability. Advancements in hybrid encapsulation systems combining proteins, polysaccharides, and lipids are also expected to improve carrier stability and functional performance. Overall, continued interdisciplinary research will be essential for translating laboratory-scale encapsulation technologies into practical industrial applications.

## 6 Conclusion

Encapsulation technologies have become essential tools for improving the stability, bioavailability, and functional performance of bioactive compounds in food, nutraceutical, and pharmaceutical applications. Bioactive compounds often exhibit poor solubility, limited stability, and susceptibility to environmental degradation, which restrict their practical use in functional products. Encapsulation provides an effective approach to overcome these limitations by entrapping sensitive bioactives within protective carrier matrices that shield them from adverse environmental conditions and enable controlled release during digestion. This review highlights the importance of various encapsulating materials, including polysaccharides, proteins, and lipid-based carriers, which offer distinct structural and functional advantages for encapsulating diverse bioactive compounds. Advanced encapsulation techniques such as ionic gelation, electrospinning, complex coacervation, and liposome-assisted delivery systems have demonstrated considerable potential to enhance encapsulation efficiency, improve stability, and enable targeted delivery. Furthermore, emerging stimuli-responsive delivery systems represent a promising strategy for achieving site-specific release of bioactive compounds in response to environmental triggers such as pH, temperature, and enzymatic activity. The continued development of innovative encapsulation materials and technologies will play a critical role in advancing the formulation of functional foods and nutraceuticals.

## References

[1] Rezagholizade-Shirvan A Soltani M Shokri S Radfar R Arab M Shamloo E. Bioactive compound encapsulation: characteristics, applications in food systems, and implications for human health. Food Chem. (2024) 24:101953. doi: 10.1016/j.fochx.2024.101953 PMC11584689 39582652

[2] Tripathy S Rout S Das RS Tiwari BK Garcia-Vaquero M Srivastav PP. A comprehensive review on algal bioactive compounds with emphasize on its allergenicity reduction, health benefits and applications in food industry. Food Biosci. (2026) 76:108285. doi: 10.1016/j.fbio.2026.108285

[3] Shahidi F Pan Y. Influence of food matrix and food processing on the chemical interaction and bioaccessibility of dietary phytochemicals: a review. Crit Rev Food Sci Nutr. (2022) 62:6421–45. doi: 10.1080/10408398.2021.1901650, 33787422

[4] Ubeyitogullari A Ahmadzadeh S Kandhola G Kim JW. Polysaccharide-based porous biopolymers for enhanced bioaccessibility and bioavailability of bioactive food compounds: challenges, advances, and opportunities. Compr Rev Food Sci Food Saf. (2022) 21:4610–39. doi: 10.1111/1541-4337.13049, 36199178

[5] Gali L Pirozzi A Donsì F. Biopolymer-and lipid-based carriers for the delivery of plant-based ingredients. Pharmaceutics. (2023) 15:927. doi: 10.3390/pharmaceutics15030927, 36986788 PMC10051097

[6] Frosi I Ferron L Colombo R Papetti A. Natural carriers: recent advances in their use to improve the stability and bioaccessibility of food active compounds. Crit Rev Food Sci Nutr. (2024) 64:5700–18. doi: 10.1080/10408398.2022.2157371, 36533404

[7] Kumari S Debbarma R Hussain S. Encapsulation strategies for enhancing the stability and shelf life of citrus bioactive compounds. Eur Food Res Technol. (2025) 251:3465–87. doi: 10.1007/s00217-025-04868-x

[8] Milinčić DD Salević-Jelić AS Kostić AŽ Stanojević SP Nedović V Pešić MB. Food nanoemulsions: how simulated gastrointestinal digestion models, nanoemulsion, and food matrix properties affect bioaccessibility of encapsulated bioactive compounds. Crit Rev Food Sci Nutr. (2024) 64:8091–113. doi: 10.1080/10408398.2023.2195519, 37021463

[9] Tripathy S Verma DK Gupta AK Srivastav PP Patel AR González MLC . Nanoencapsulation of biofunctional components as a burgeoning nanotechnology-based approach for functional food development: a review. Biocatal Agric Biotechnol. (2023) 53:102890. doi: 10.1016/j.bcab.2023.102890

[10] Rakshit M Tripathy S Srivastav PP. "Encapsulation of natural polyphenols for food applications". In: Novel Processing Methods for Plant-Based Health Foods. (eds). Goyal MR Veena N Watharkar RB. New York: Apple Academic Press (2023). p. 123–62.

[11] Prabhakar P Tripathy S Verma DK Singh S Thakur M Singh AK . Trends and advances in liposome formulation technology with an emphasis on ensuring safety and quality in food and drug applications. Food Biosci. (2025) 69:106913. doi: 10.1016/j.fbio.2025.106913

[12] Loffredi E Alamprese C. Digestion fate and food applications of emulsions as delivery systems for bioactive compounds: challenges and perspectives. Food Rev Intl. (2024) 40:2103–27. doi: 10.1080/87559129.2023.2249098

[13] Tripathy S Verma DK Thakur M Patel AR Srivastav PP Singh S . Encapsulated food products as a strategy to strengthen immunity against COVID-19. Front Nutr. (2021) 8:673174. doi: 10.3389/fnut.2021.673174, 34095193 PMC8175800

[14] Singh SM Tripathy S Ghodki BM Srivastav PP. Optimization of wall material composition for encapsulating bioactive compounds from Giloy (Tinospora cordifolia) stems. J Food Process Preserv. (2025) 2025:6459848. doi: 10.1155/jfpp/6459848

[15] Jia Y Wang C Khalifa I Zhu Y Wang Z Chen H . Pectin: a review with recent advances in the emerging revolution and multiscale evaluation approaches of its emulsifying characteristics. Food Hydrocoll. (2024) 157:110428. doi: 10.1016/j.foodhyd.2024.110428

[16] Montoya-Yepes DF Jimenez-Rodriguez AA Aldana-Porras AE Velasquez-Holguin LF Mendez-Arteaga JJ Murillo-Arango W. Starches in the encapsulation of plant active ingredients: state of the art and research trends. Polym Bull. (2024) 81:135–63. doi: 10.1007/s00289-023-04724-6

[17] Jiang L Zhang Z Qiu C Wen J. A review of whey protein-based bioactive delivery systems: design, fabrication, and application. Foods. (2024) 13:2453. doi: 10.3390/foods13152453, 39123644 PMC11312236

[18] Tan Y Zi Y Peng J Shi C Zheng Y Zhong J. Gelatin as a bioactive nanodelivery system for functional food applications. Food Chem. (2023) 423:136265. doi: 10.1016/j.foodchem.2023.136265, 37167667

[19] Riquelme N Alarcón-Moyano J Garrido-Miranda K Díaz-Calderón P Arancibia C Burgos-Díaz C. A current review of edible Pickering emulsion-based delivery systems to enhance stability and bioaccessibility of lipophilic bioactive compounds. Food Rev Intl. (2025) 42:1–34. doi: 10.1080/87559129.2025.2518477

[20] Sethunga M Rangani SC Munaweera I Ranaweera KKDS. Hybrid nanoencapsulation systems: integrating natural polymers with synthetic nanomaterials for enhanced delivery of bioactive compounds in functional foods. Nanoscale Adv. (2025) 7:7867–90. doi: 10.1039/D5NA00751H 41220488 PMC12598503

[21] Chavda VP Patel AB Mistry KJ Suthar SF Wu ZX Chen ZS . Nano-drug delivery systems entrapping natural bioactive compounds for cancer: recent progress and future challenges. Front Oncol. (2022) 12:867655. doi: 10.3389/fonc.2022.867655, 35425710 PMC9004605

[22] Palai S Priyadarshini A Sadangi S. "Challenges in effective delivery and utilization of bioactive compounds". In: Harnessing Nanoencapsulation: Valorization of Bioactive Compounds for Health and Beyond. (eds). Ahmad Wani S Rashid Naik H. Springer Nature Switzerland: Cham (2025). p. 53–72.

[23] Protsak IS Morozov YM. Fundamentals and advances in stimuli-responsive hydrogels and their applications: a review. Gels. (2025) 11:30. doi: 10.3390/gels11010030, 39852001 PMC11765116

[24] Mashele SS. Stimuli-responsive, cell-mediated drug delivery systems: engineering smart cellular vehicles for precision therapeutics. Pharmaceutics. (2025) 17:1082. doi: 10.3390/pharmaceutics17081082, 40871101 PMC12389507

[25] Meng Y Qiu C Li X McClements DJ Sang S Jiao A . Polysaccharide-based nano-delivery systems for encapsulation, delivery, and pH-responsive release of bioactive ingredients. Crit Rev Food Sci Nutr. (2024) 64:187–201. doi: 10.1080/10408398.2022.2105800, 35930011

[26] Yang E Jung HS Chang PS. Stimuli-responsive polymer-complexed liposome nanocarrier provides controlled release of biomolecules. Food Hydrocoll. (2022) 125:107397. doi: 10.1016/j.foodhyd.2021.107397

[27] Xu Y Guo J Wei Z Xue C. Cellulose-based delivery systems for bioactive ingredients: a review. Int J Biol Macromol. (2025) 299:140072. doi: 10.1016/j.ijbiomac.2025.140072 39842568

[28] Zabot GL Schaefer Rodrigues F Polano Ody L Vinícius Tres M Herrera E Palacin H . Encapsulation of bioactive compounds for food and agricultural applications. Polymers. (2022) 14:4194. doi: 10.3390/polym14194194, 36236142 PMC9571964

[29] Shishir MRI Xie L Sun C Zheng X Chen W. Advances in micro and nano-encapsulation of bioactive compounds using biopolymer and lipid-based transporters. Trends Food Sci Technol. (2018) 78:34–60. doi: 10.1016/j.tifs.2018.05.018

[30] Falcão LDS Coelho DB Veggi PC Campelo PH Albuquerque PM de Moraes MA. Starch as a matrix for incorporation and release of bioactive compounds: fundamentals and applications. Polymers. (2022) 14:2361. doi: 10.3390/polym14122361, 35745937 PMC9228233

[31] Bai Y Shi YC. Chemical structures in pyrodextrin determined by nuclear magnetic resonance spectroscopy. Carbohydr Polym. (2016) 151:426–33. doi: 10.1016/j.carbpol.2016.05.058, 27474585

[32] Hoti G Matencio A Rubin Pedrazzo A Cecone C Appleton SL Khazaei Monfared Y . Nutraceutical concepts and dextrin-based delivery systems. Int J Mol Sci. (2022) 23:4102. doi: 10.3390/ijms23084102, 35456919 PMC9031143

[33] Selamassakul O Kaisangsri N Sonklin C Kaprasob R Uthairatanakij A Laohakunjit N. Effects of cluster dextrin encapsulation on the properties and antioxidant stability of fractionated Riceberry protein hydrolysate powder prepared by bromelain. Food Chem. (2024) 439:138161. doi: 10.1016/j.foodchem.2023.138161, 38070233

[34] Xiao Z Xia J Zhao Q Niu Y Zhao D. Maltodextrin as wall material for microcapsules: a review. Carbohydr Polym. (2022) 298:120113. doi: 10.1016/j.carbpol.2022.120113, 36241287

[35] Sanchez V Baeza R Galmarini MV Zamora MC Chirife J. Freeze-drying encapsulation of red wine polyphenols in an amorphous matrix of maltodextrin. Food Bioprocess Technol. (2013) 6:1350–4. doi: 10.1007/s11947-011-0654-z

[36] Gandia-Herrero F Jimenez-Atienzar M Cabanes J Garcia-Carmona F Escribano J. Stabilization of the bioactive pigment of Opuntia fruits through maltodextrin encapsulation. J Agric Food Chem. (2010) 58:10646–52. doi: 10.1021/jf101695f, 20812722

[37] Kfoury M Auezova L Greige-Gerges H Fourmentin S. Encapsulation in cyclodextrins to widen the applications of essential oils. Environ Chem Lett. (2019) 17:129–43. doi: 10.1007/s10311-018-0783-y

[38] Trygg J Yildir E Kolakovic R Sandler N Fardim P. Anionic cellulose beads for drug encapsulation and release. Cellulose. (2014) 21:1945–55. doi: 10.1007/s10570-014-0253-z

[39] Vukoja J Buljeta I Pichler A Šimunović J Kopjar M. Formulation and stability of cellulose-based delivery systems of raspberry phenolics. PRO. (2021) 9:90. doi: 10.3390/pr9010090

[40] Naqash F Masoodi FA Rather SA Wani SM Gani A. Emerging concepts in the nutraceutical and functional properties of pectin—a review. Carbohydr Polym. (2017) 168:227–39. doi: 10.1016/j.carbpol.2017.03.058, 28457445

[41] Rosales TKO Fabi JP. Pectin-based nanoencapsulation strategy to improve the bioavailability of bioactive compounds. Int J Biol Macromol. (2023) 229:11–21. doi: 10.1016/j.ijbiomac.2022.12.292, 36586647

[42] Shishir MRI Karim N Gowd V Xie J Zheng X Chen W. Pectin-chitosan conjugated nanoliposome as a promising delivery system for neohesperidin: characterization, release behavior, cellular uptake, and antioxidant property. Food Hydrocoll. (2019) 95:432–44. doi: 10.1016/j.foodhyd.2019.04.059

[43] Noreen A Akram J Rasul I Mansha A Yaqoob N Iqbal R . Pectins functionalized biomaterials; a new viable approach for biomedical applications: a review. Int J Biol Macromol. (2017) 101:254–72. doi: 10.1016/j.ijbiomac.2017.03.029 28300586

[44] Khotimchenko M. Pectin polymers for colon-targeted antitumor drug delivery. Int J Biol Macromol. (2020) 158:1110–24. doi: 10.1016/j.ijbiomac.2020.05.002, 32387365

[45] Shah P. "Polymers in food". In: Polymer Science and Innovative Applications. (eds). Al Ali AlMaadeed M. Ponnamma D. Carignano M. A.. Elsevier (2020). p. 567–92.

[46] Akbari-Alavijeh S Shaddel R Jafari SM. Encapsulation of food bioactives and nutraceuticals by various chitosan-based nanocarriers. Food Hydrocoll. (2020) 105:105774. doi: 10.1016/j.foodhyd.2020.105774

[47] Paula HC Sombra FM Abreu FO Paul R. Lippia sidoides essential oil encapsulation by Angico gum/chitosan nanoparticles. J Braz Chem Soc. (2010) 21:2359–66. doi: 10.1590/S0103-50532010001200025

[48] Liang J Yan H Puligundla P Gao X Zhou Y Wan X. Applications of chitosan nanoparticles to enhance absorption and bioavailability of tea polyphenols: a review. Food Hydrocoll. (2017) 69:286–92. doi: 10.1016/j.foodhyd.2017.01.041

[49] Yin C Cheng L Zhang X Wu Z. Nanotechnology improves delivery efficiency and bioavailability of tea polyphenols. J Food Biochem. (2020) 44:e13380. doi: 10.1111/jfbc.13380, 32667062

[50] Wang W Jung J Zhao Y. Chitosan-cellulose nanocrystal microencapsulation to improve encapsulation efficiency and stability of entrapped fruit anthocyanins. Carbohydr Polym. (2017) 157:1246–53. doi: 10.1016/j.carbpol.2016.11.005, 27987829

[51] Hossain F Follett P Salmieri S Vu KD Fraschini C Lacroix M. Antifungal activities of combined treatments of irradiation and essential oils (EOs) encapsulated chitosan nanocomposite films in in vitro and in situ conditions. Int J Food Microbiol. (2019) 295:33–40. doi: 10.1016/j.ijfoodmicro.2019.02.009, 30784857

[52] Hu Q Luo Y. Chitosan-based nanocarriers for encapsulation and delivery of curcumin: a review. Int J Biol Macromol. (2021) 179:125–35. doi: 10.1016/j.ijbiomac.2021.02.216, 33667554

[53] Karim A Rehman A Feng J Noreen A Assadpour E Kharazmi MS . Alginate-based nanocarriers for the delivery and controlled-release of bioactive compounds. Adv Colloid Interf Sci. (2022) 307:102744. doi: 10.1016/j.cis.2022.102744, 35878506

[54] Fernando IPS Lee W Han EJ Ahn G. Alginate-based nanomaterials: fabrication techniques, properties, and applications. Chem Eng J. (2020) 391:123823. doi: 10.1016/j.cej.2019.123823

[55] Li J Hao X Gan W van Loosdrecht MC Wu Y. Controlling factors and involved mechanisms on forming alginate like extracellular polymers in flocculent sludge. Chem Eng J. (2022) 439:135792. doi: 10.1016/j.cej.2022.135792

[56] Gómez-Mascaraque LG Martínez-Sanz M Hogan SA López-Rubio A Brodkorb A. Nano-and microstructural evolution of alginate beads in simulated gastrointestinal fluids. Impact of M/G ratio, molecular weight and pH. Carbohydr Polym. (2019) 223:115121. doi: 10.1016/j.carbpol.2019.115121, 31427011

[57] Morrish C Teimouri S Istivan T Kasapis S. Molecular characterisation of hot moulded alginate gels as a delivery vehicle for the release of entrapped caffeine. Food Hydrocoll. (2020) 109:106142. doi: 10.1016/j.foodhyd.2020.106142

[58] Dong X Du S Deng Q Tang H Yang C Wei F . Study on the antioxidant activity and emulsifying properties of flaxseed gum-whey protein isolate conjugates prepared by Maillard reaction. Int J Biol Macromol. (2020) 153:1157–64. doi: 10.1016/j.ijbiomac.2019.10.245, 31760021

[59] Busch VM Pereyra-Gonzalez A Šegatin N Santagapita PR Ulrih NP Buera MDP. Propolis encapsulation by spray drying: characterization and stability. LWT. (2017) 75:227–35. doi: 10.1016/j.lwt.2016.08.055

[60] Abaee A Mohammadian M Jafari SM. Whey and soy protein-based hydrogels and nano-hydrogels as bioactive delivery systems. Trends Food Sci Technol. (2017) 70:69–81. doi: 10.1016/j.tifs.2017.10.011

[61] Morales EAC Doost AS Velazquez G Van der Meeren P. Comparison of low-and high-methoxyl pectin for the stabilization of whey protein isolate as carrier for lutein. Food Hydrocoll. (2021) 113:106458. doi: 10.1016/j.foodhyd.2020.106458

[62] Gunasekaran S Ko S Xiao L. Use of whey proteins for encapsulation and controlled delivery applications. J Food Eng. (2007) 83:31–40. doi: 10.1016/j.jfoodeng.2006.11.001

[63] Liu K Zha XQ Shen WD Li QM Pan LH Luo JP. The hydrogel of whey protein isolate coated by lotus root amylopectin enhance the stability and bioavailability of quercetin. Carbohydr Polym. (2020) 236:116009. doi: 10.1016/j.carbpol.2020.116009, 32172837

[64] Acuña-Avila PE Cortes-Camargo S Jiménez-Rosales A. Properties of micro and nano casein capsules used to protect the active components: a review. Int J Food Prop. (2021) 24:1132–47. doi: 10.1080/10942912.2021.1953069

[65] Lelis CA Galvan D Conte-Junior CA. Nanocarriers for β-carotene based on milk protein. Food Bioprocess Technol. (2023) 16:43–67. doi: 10.1007/s11947-022-02868-3

[66] Ghatak D Iyyaswami R. Selective encapsulation of quercetin from dry onion peel crude extract in reassembled casein particles. Food Bioprod Process. (2019) 115:100–9. doi: 10.1016/j.fbp.2019.03.003

[67] Peñalva R Morales J González-Navarro CJ Larrañeta E Quincoces G Peñuelas I . Increased oral bioavailability of resveratrol by its encapsulation in casein nanoparticles. Int J Mol Sci. (2018) 19:2816. doi: 10.3390/ijms19092816, 30231546 PMC6163610

[68] Zhao Z Corredig M. Changes in the physico-chemical properties of casein micelles in the presence of sodium chloride in untreated and concentrated milk protein. Dairy Sci Technol. (2015) 95:87–99. doi: 10.1007/s13594-014-0200-7

[69] Wang X Gao S Yun S Zhang M Peng L Li Y . Microencapsulating alginate-based polymers for probiotics delivery systems and their application. Pharmaceuticals. (2022) 15:644. doi: 10.3390/ph15050644, 35631470 PMC9144165

[70] Akbarbaglu Z Peighambardoust SH Sarabandi K Jafari SM. Spray drying encapsulation of bioactive compounds within protein-based carriers; different options and applications. Food Chem. (2021) 359:129965. doi: 10.1016/j.foodchem.2021.129965, 33975145

[71] Xiao J. (ed.). "The collagen Suprafamily". In: Collagen Mimetic Peptides and Their Biophysical Characterization. Springer Nature Singapore: Singapore (2024). p. 1–24.

[72] Hani NM Torkamani AE Azarian MH Mahmood KW Ngalim SH. Characterisation of electrospun gelatine nanofibres encapsulated with *Moringa oleifera* bioactive extract. J Sci Food Agric. (2017) 97:3348–58. doi: 10.1002/jsfa.8185, 27981649

[73] Ganeson K Razifah MR Mubarak A Kam A Vigneswari S Ramakrishna S. Improved functionality of cinnamon oil emulsion-based gelatin films as potential edible packaging film for wax apple. Food Biosci. (2022) 47:101638. doi: 10.1016/j.fbio.2022.101638

[74] Devarajan A Mudgil P Aldhaheri F Hamed F Dhital S Maqsood S. Camel milk-derived probiotic strains encapsulated in camel casein and gelatin complex microcapsules: stability against thermal challenge and simulated gastrointestinal digestion conditions. J Dairy Sci. (2022) 105:1862–77. doi: 10.3168/jds.2021-20745, 34998543

[75] Łopusiewicz Ł Kwiatkowski P Drozłowska E Trocer P Kostek M Śliwiński M . Preparation and characterization of carboxymethyl cellulose-based bioactive composite films modified with fungal melanin and carvacrol. Polymers. (2021) 13:499. doi: 10.3390/polym13040499, 33562865 PMC7914822

[76] Sugino H Nitoda T Juneja LR. "General chemical composition of hen eggs". In: Hen Eggs. (eds). Yamamoto T Raj Juneja L Hatta H Kim M. Boca Raton: CRC Press. (2018). p. 13–24.

[77] Zhang Q Chen Y Geng F Shen X. Characterization of spray-dried microcapsules of paprika oleoresin induced by ultrasound and high-pressure homogenization: physicochemical properties and storage stability. Molecules. (2023) 28:7075. doi: 10.3390/molecules28207075, 37894554 PMC10609558

[78] Zhang Y Guo Y Liu F Luo Y. Recent development of egg protein fractions and individual proteins as encapsulant materials for delivery of bioactives. Food Chem. (2023) 403:134353. doi: 10.1016/j.foodchem.2022.134353, 36179637

[79] Dong X Zhang YQ. An insight on egg white: from most common functional food to biomaterial application. J Biomed Mater Res B Appl Biomater. (2021) 109:1045–58. doi: 10.1002/jbm.b.34768, 33252178

[80] Liu X Zhang M Zhou X Wan M Cui A Xiao B . Research advances in Zein-based nano-delivery systems. Front Nutr. (2024) 11:1379982. doi: 10.3389/fnut.2024.1379982, 38798768 PMC11119329

[81] Yao L Jiang A Chen L. Characterization of ethanol-induced egg white gel and transportation of active nutraceuticals. Lwt. (2020) 130:109530. doi: 10.1016/j.lwt.2020.109530

[82] Shanshan W Meigui H Chunyang L Zhi C Li C Wuyang H . Fabrication of ovalbumin-burdock polysaccharide complexes as interfacial stabilizers for nanostructured lipid carriers: effects of high-intensity ultrasound treatment. Food Hydrocoll. (2021) 111:106407. doi: 10.1016/j.foodhyd.2020.106407

[83] Di Giorgio L Salgado PR Mauri AN. Fish oil encapsulated in soy protein particles by lyophilization. Effect of drying process. J Sci Food Agric. (2022) 102:206–13. doi: 10.1002/jsfa.11347, 34061354

[84] Rout S Srivastav PP. Modification of soy protein isolate and pea protein isolate by high voltage dielectric barrier discharge (DBD) atmospheric cold plasma: comparative study on structural, rheological and techno-functional characteristics. Food Chem. (2024) 447:138914. doi: 10.1016/j.foodchem.2024.138914, 38460320

[85] Tavernier I Heyman B Van der Meeren P Ruyssen T Dewettinck K. Oil powders stabilized with soy protein used to prepare oil-in-fat dispersions. J Food Eng. (2019) 244:136–41. doi: 10.1016/j.jfoodeng.2018.09.029

[86] Martínez-López AL Pangua C Reboredo C Campión R Morales-Gracia J Irache JM. Protein-based nanoparticles for drug delivery purposes. Int J Pharm. (2020) 581:119289. doi: 10.1016/j.ijpharm.2020.119289, 32243968

[87] Souza ACP Gurak PD Marczak LDF. Maltodextrin, pectin and soy protein isolate as carrier agents in the encapsulation of anthocyanins-rich extract from Jaboticaba pomace. Food Bioprod Process. (2017) 102:186–94. doi: 10.1016/j.fbp.2016.12.012

[88] Fan L Lu Y Ouyang XK Ling J. Development and characterization of soybean protein isolate and fucoidan nanoparticles for curcumin encapsulation. Int J Biol Macromol. (2021) 169:194–205. doi: 10.1016/j.ijbiomac.2020.12.086, 33340634

[89] Fathi M Donsi F McClements DJ. Protein-based delivery systems for the nanoencapsulation of food ingredients. Compr Rev Food Sci Food Saf. (2018) 17:920–36. doi: 10.1111/1541-4337.12360, 33350116

[90] Tadele DT Mekonnen TH. Co-encapsulation of quercetin and α-Tocopherol bioactives in zein nanoparticles: synergistic interactions, stability, and controlled release. ACS Appl Polym Mater. (2024) 6:3767–77. doi: 10.1021/acsapm.3c03000

[91] Oleandro E Stanzione M Buonocore GG Lavorgna M. Zein-based nanoparticles as active platforms for sustainable applications: recent advances and perspectives. Nano. (2024) 14:414. doi: 10.3390/nano14050414, 38470745 PMC10934072

[92] Islam F Amer Ali Y Imran A Afzaal M Zahra SM Fatima M . Vegetable proteins as encapsulating agents: recent updates and future perspectives. Food Sci Nutr. (2023) 11:1705–17. doi: 10.1002/fsn3.3234, 37051354 PMC10084973

[93] Cao J Fan G Wang C Lu B. Self-assembling wheat gluten peptide nanoparticles: Pterostilbene encapsulation and interaction mechanism. Food Hydrocoll. (2024) 162, 110960. doi: 10.1016/j.foodhyd.2024.110960

[94] Fox GP. "Chemical composition in barley grains and malt quality". In: Genetics and Improvement of Barley Malt Quality. eds. Zhang G Li C. Berlin: Springer Berlin Heidelberg (2009). p. 63–98.

[95] Jaeger A Zannini E Sahin AW Arendt EK. Barley protein properties, extraction and applications, with a focus on brewers’ spent grain protein. Foods. (2021) 10:1389. doi: 10.3390/foods10061389, 34208463 PMC8234785

[96] Meira ACFDO Morais LCD Figueiredo JDA Veríssimo LAA Botrel DA Resende JVD. Microencapsulation of β-carotene using barley residue proteins from beer waste as coating material. J Microencapsul. (2023) 40:171–85. doi: 10.1080/02652048.2023.2183277, 36803148

[97] Khatun A Waters DLE Liu L. The impact of rice protein on in vitro rice starch digestibility. Food Hydrocoll. (2020) 109:106072. doi: 10.1016/j.foodhyd.2020.106072

[98] Kelemen V Pichler A Ivić I Buljeta I Šimunović J Kopjar M. Brown rice proteins as delivery system of phenolic and volatile compounds of raspberry juice. Int J Food Sci Technol. (2022) 57:1866–74. doi: 10.1111/ijfs.15023

[99] Kopjar M Buljeta I Ćorković I Pichler A Šimunović J. Adsorption of quercetin on brown rice and almond protein matrices: effect of quercetin concentration. Foods. (2022) 11:793. doi: 10.3390/foods11060793, 35327216 PMC8947628

[100] Janssen F Pauly A Rombouts I Jansens KJ Deleu LJ Delcour JA. Proteins of amaranth (Amaranthus spp.), buckwheat (Fagopyrum spp.), and quinoa (Chenopodium spp.): a food science and technology perspective. Compr Rev Food Sci Food Saf. (2017) 16:39–58. doi: 10.1111/1541-4337.12240, 33371541

[101] Hadidi M Boostani S Jafari SM. Pea proteins as emerging biopolymers for the emulsification and encapsulation of food bioactives. Food Hydrocoll. (2022) 126:107474. doi: 10.1016/j.foodhyd.2021.107474

[102] Blanco-Padilla A López-Rubio A Loarca-Piña G Gómez-Mascaraque LG Mendoza S. Characterization, release and antioxidant activity of curcumin-loaded amaranth-pullulan electrospun fibers. LWT Food Sci Technol. (2015) 63:1137–44. doi: 10.1016/j.lwt.2015.03.081

[103] Thy LTM Duy HK Dat NM. Applications of lecithin in emulsion stabilization and advanced delivery systems in cosmetics: a mini-review. Results Surf Interfaces. (2025) 19:100543. doi: 10.1016/j.rsurfi.2025.100543

[104] Tripathy S Srivastav PP. Encapsulation of *Centella asiatica* leaf extract in liposome: study on structural stability, degradation kinetics and fate of bioactive compounds during storage. Food Chem Adv. (2023) 2:100202. doi: 10.1016/j.focha.2023.100202

[105] Lazăr AR Pușcaș A Tanislav AE Mureșan V. Bioactive compounds delivery and bioavailability in structured edible oils systems. Compr Rev Food Sci Food Saf. (2024) 23:e70020. doi: 10.1111/1541-4337.70020, 39437192

[106] Zhou H Zheng B McClements DJ. Encapsulation of lipophilic polyphenols in plant-based nanoemulsions: impact of carrier oil on lipid digestion and curcumin, resveratrol and quercetin bioaccessibility. Food Funct. (2021) 12:3420–32. doi: 10.1039/D1FO00275A, 33900331

[107] Tripathy S Srivastav PP. Sustainable liposomal delivery of *Centella asiatica* polyphenols: β-sitosterol stabilization, LC-MS/MS profiling, and simulated release study. Sustain Food Technol. (2025) 3:1053–63. doi: 10.1039/D5FB00127G

[108] Liu X Song Z Tian W Abdullah Huang Q Chen M Xiao J. Advancements in lipid-based delivery systems for functional foods: a comprehensive review of literature and patent trends. Crit Rev Food Sci Nutr. (2025) 65:2456–72. doi: 10.1080/10408398.2024.2343415, 38693696

[109] Khanvilkar AM Ranveer RC Sahoo AK. Carrier materials for encapsulation of bio-active components of food. Int J Pharm Sci Rev Res. (2016) 40:62–73.

[110] Pech-Canul ADLC Ortega D García-Triana A González-Silva N Solis-Oviedo RL. A brief review of edible coating materials for the microencapsulation of probiotics. Coatings. (2020) 10:197. doi: 10.3390/coatings10030197

[111] Su X Toublan F Yin Y Cadwallader KR. "Fats and waxes in microencapsulation of food ingredients". In: Microencapsulation in the Food Industry. (ed.) Sobel R, Academic Press (2023). p. 325–42.

[112] Soleimanian Y Goli SAH Shirvani A Elmizadeh A Marangoni AG. Wax-based delivery systems: preparation, characterization, and food applications. Compr Rev Food Sci Food Saf. (2020) 19:2994–3030. doi: 10.1111/1541-4337.12614, 33337056

[113] Hashim SB Tahir HE Mahdi AA Al-Maqtari QA Shishir MRI Mahunu GK . Enhancing the functionality of the Origanum compactum essential oil capsules by combining sugarcane wax with various biopolymers. J Food Meas Charact. (2025) 19:833–49. doi: 10.1007/s11694-024-02915-x

[114] Kumar S Cherwoo L Puri N Sharma A Thombare N Bhondekar AP. "Shellac: a natural lipid polymer for food safety and quality monitoring". In: Nanotechnology Applications for Food Safety and Quality Monitoring. (eds). Sharma A Vijayakumar PS Prabhakar Er. PK Kumar R. Academic Press (2023). p. 135–54.

[115] Sharma S Samrat Goyal P Dhingra K Singh A Sarkar A Poddar D. Shellac: bridging the gap between chemistry and sustainability—a comprehensive review of its multifunctional coating applications for food, drug, and paper packaging. J Macromol Sci A. (2024) 61:691–723. doi: 10.1080/10601325.2024.2400510

[116] Zhang L Zhang J Zhang J Huang X Shi J. Advances in pH-responsive release Technologies in Food System: mechanisms, strategies, application forms and future directions. Foods. (2025) 14:3896. doi: 10.3390/foods14223896 41300054 PMC12651379

[117] Baek J Ramasamy M Cho DG Soo CCC Kapar S Lee JY . A new approach for the encapsulation of *Saccharomyces cerevisiae* using shellac and cellulose nanocrystals. Food Hydrocoll. (2023) 134:108079. doi: 10.1016/j.foodhyd.2022.108079

[118] Yin M Zhang Q Zhong F. Construction of double network gel for co-encapsulation of probiotics and capsaicin: enhanced the physicochemical stability and controlled release. Food Biosci. (2024) 58:103715. doi: 10.1016/j.fbio.2024.103715

[119] Shu D Liu Y Xu J Yuan Y. A review of shellac-based carrier design for food application: from the perspective of core materials. LWT. (2025) 232:118475. doi: 10.1016/j.lwt.2025.118475

[120] Zulfakar MH Pubadi H Ibrahim SI Hairul NM. Medium-chain triacylglycerols (MCTs) and their fractions in drug delivery systems: a systematic review. J Oleo Sci. (2024) 73:293–310. doi: 10.5650/jos.ess23204, 38432994

[121] Guo Y Song Z Cao Y Xiao J. Impact of cocoa butter and medium chain triglycerides ratios on processing stability, supersaturation, and digestive properties of curcumin-loaded nanostructured lipid carriers. LWT. (2024) 197:115895. doi: 10.1016/j.lwt.2024.115895

[122] Yang Z Guo Y Zeng C Sun F Wang Z Zhang W . Encapsulation and characterization of ω-3 medium-and long-chain triacylglycerols microencapsulated with different proteins as wall materials. Food Chem. (2024) 22:101363. doi: 10.1016/j.fochx.2024.101363 PMC11052903 38681229

[123] Narwal RS Rustagi S Pandey VK. Development and characterization of β-carotene-enriched oleogels based on beeswax–medium-chain triglyceride oils. Nat. Res. Human Health. (2025) 5:499–509. doi: 10.53365/nrfhh/200472

[124] Hasnain MS Barik H Sahoo RN Pattanayak P Panda BB Nayak AK. "Ionotropic cross-linking of biopolymers: basics and mechanisms". In: Ionotropic Cross-Linking of Biopolymers. (eds.) Kumar NA Saquib HM. Elsevier (2024). p. 3–31.

[125] Wang B LvYe J Yang S Shi Y Chen Q. Critical review of food colloidal delivery system for bioactive compounds: physical characterization and application. Foods. (2024) 13:2596. doi: 10.3390/foods13162596, 39200523 PMC11353541

[126] Wurm F Rietzler B Pham T Bechtold T. Multivalent ions as reactive crosslinkers for biopolymers—a review. Molecules. (2020) 25:1840. doi: 10.3390/molecules25081840, 32316293 PMC7221734

[127] Chan LW Lee HY Heng PW. Mechanisms of external and internal gelation and their impact on the functions of alginate as a coat and delivery system. Carbohydr Polym. (2006) 63:176–87. doi: 10.1016/j.carbpol.2005.07.033

[128] Özgen S Ertop MH Ertop U. Formulation and evaluation of Propolis-loaded alginate capsules by ionic gelation technique. Food Bioprocess Technol. (2025) 18:7531–43. doi: 10.1007/s11947-025-03889-4

[129] de Oliveira JM de Jesus MS Cabral AV de Andrade Wartha ERS Narain N Pagani AAC. Characterization of microcapsules obtained from lemon balm extract (*Melissa officinalis* L.) by the ionic gelation process. Plant Foods Hum Nutr. (2025) 80:42. doi: 10.1007/s11130-025-01295-x, 39836260

[130] Santos AP Andreola K Alvim ID de Moura SCSR Hubinger MD. Microencapsulation of Pitanga extract (*Eugenia uniflora* L.) by ionic gelation: effect of wall material and fluidized bed drying. Food Res Int. (2025) 209:116304. doi: 10.1016/j.foodres.2025.116304, 40253150

[131] Wani KM Uppaluri RV. Efficacy of ionic gelation based encapsulation of bioactives from papaya leaf extract: characterization and storage stability. Biomass Convers Biorefinery. (2024) 14:19911–28. doi: 10.1007/s13399-023-03956-w

[132] Herman-Lara E Rivera-Abascal I Gallegos-Marín I Martínez-Sánchez CE. Encapsulation of hydroalcoholic extracts of *Moringa oleifera* seed through ionic gelation. LWT. (2024) 203:116368. doi: 10.1016/j.lwt.2024.116368

[133] Kurtulbaş E Albarri R Torun M Şahin S. Encapsulation of *Moringa oleifera* leaf extract in chitosan-coated alginate microbeads produced by ionic gelation. Food Biosci. (2022) 50:102158. doi: 10.1016/j.fbio.2022.102158

[134] Martinović J Lukinac J Jukić M Ambrus R Planinić M Šelo G . In vitro bioaccessibility assessment of phenolic compounds from encapsulated grape pomace extract by ionic gelation. Molecules. (2023) 28:5285. doi: 10.3390/molecules28135285, 37446946 PMC10343682

[135] Petraitytė S Šipailienė A. Enhancing encapsulation efficiency of alginate capsules containing lactic acid bacteria by using different divalent cross-linkers sources. Lwt. (2019) 110:307–15. doi: 10.1016/j.lwt.2019.01.065

[136] Yun P Devahastin S Chiewchan N. Microstructures of encapsulates and their relations with encapsulation efficiency and controlled release of bioactive constituents: a review. Compr Rev Food Sci Food Saf. (2021) 20:1768–99. doi: 10.1111/1541-4337.12701, 33527760

[137] Tripathy S Srivastav PP. Encapsulation of food bioactive compounds using Electrohydrodynamic techniques: from fundamentals to industrial applications. Front Food Sci Technol. (2026) 6:1731969. doi: 10.3389/frfst.2026.1731969

[138] Jansen ET da Cruz EP Fonseca LM Radünz M Camargo TM Dias ARG . Electrospun encapsulation of grape pomace extract: in vitro antioxidant, anti-inflammatory, and antihyperglycemic properties. J Sci Food Agric. (2026) 106:4247–55. doi: 10.1002/jsfa.70506, 41645611 PMC13067086

[139] Mohagheghian R Pezeshki A Ghanbarzadeh B Mohammadi M Aboudzadeh MA. Electrospun Gelatin/OSA-starch Nanofibers for encapsulation and controlled release of sage extract. J Appl Polym Sci. (2026) 143:70466. doi: 10.1002/app.70466

[140] Saghaee R Ariaii P. Development of electrospun whey protein isolate nanofiber mat for omega-3 nanoencapsulation: microstructural and physical property analysis. Int J Biol Macromol. (2025) 301:140273. doi: 10.1016/j.ijbiomac.2025.140273, 39870276

[141] İnan-Çınkır N Ağçam E Altay F Akyıldız A. Emulsion electrospinning of zein nanofibers with carotenoid microemulsion: optimization, characterization and fortification. Food Chem. (2024) 430:137005. doi: 10.1016/j.foodchem.2023.137005, 37527575

[142] Alrosan M Al-Rabadi N Alu’datt MH Al-Qaisi A Al-Shunnaq EAE Abu-Khalaf N . Complex coacervation of plant-based proteins and polysaccharides: sustainable encapsulation techniques for bioactive compounds. Food Eng Rev. (2025) 17:1059–82. doi: 10.1007/s12393-025-09408-7

[143] Pongpairoj P Sae-tan S Iwamoto S Peanparkdee M. Microencapsulation of bioactive compounds from tender aromatic coconut mesocarp via complex coacervation using soy protein and polysaccharides. J Food Eng. (2026) 402:112694. doi: 10.1016/j.jfoodeng.2025.112694

[144] Bispo NF Fonseca HC Araújo BRS Vieira CR Alves ÉE Pinto MS . Propolis microencapsulation by complex coacervation using whey protein and gum arabic: an approach to the assessment of the stability and controlled release of phenolic compounds. Food Sci Technol. (2026) 46:577. doi: 10.5327/fst.577

[145] Rajabi H Razavi SMA. Co-encapsulation of saffron petal and Stachys schtschegleevii extracts via complex coacervation and graphene oxide-assisted spray drying for co-delivery and stability enhancement. LWT. (2025) 223:117705. doi: 10.1016/j.lwt.2025.117705

[146] Djihad N Naima FO Petronilho S Hamid S Bedjou FNE Coimbra MA. Microencapsulation of *Citrus limon* essential oil by complex coacervation and release behavior of terpenic and derived volatile compounds. Food Hydrocoll. (2024) 152:109830. doi: 10.1016/j.foodhyd.2024.109830

[147] Ozkan G Ugur ES Capanoglu E. Ultrasonication-enabled liposomal encapsulation of Propolis extract: bioaccessibility, bioavailability, and food application. Food Bioprocess Technol. (2026) 19:189. doi: 10.1007/s11947-026-04205-4

[148] Zhang Y Gu YF Yu LF Yan LX. Whey protein-κ-carrageenan coated Mangiferin liposomes for enhanced stability and bioavailability. Int J Biol Macromol. (2026) 347:150763. doi: 10.1016/j.ijbiomac.2026.150763, 41651270

[149] Kaushik D. Krishnan H. Kumar M. Oz E. Oz F. (2024). Interdisciplinary approaches to encapsulation in food science: sustainable methods, Omics techniques, and industrial applications. In Basic Protocols in Encapsulation of Food Ingredients. (ed.) Gomez-Zavaglia A, (pp. 153–165). New York, NY: Springer US.

[150] Gunel Z. Dual-layer microencapsulation of grape skin extract via spray chilling and fluid-bed coating for targeted intestinal release. Eur Food Res Technol. (2026) 252:184. doi: 10.1007/s00217-026-05070-3

[151] Nascimento RF de França PRL Ferreira MA Kurozawa LE. Assessment of the protective potential of coated microparticles in a fluidized bed against the simulated digestion. Food Res Int. (2025) 208:116273. doi: 10.1016/j.foodres.2025.116273, 40263813

[152] Albuquerque RCV de Farias Silva CE Silva MCDS Carneiro WDS Andreola K Gama BMVD . Incorporation of agglomerated Spirulina platensis powder in yogurt: a strategy for enhancing nutritional quality and bioactive compounds. Fermentation. (2025) 11:389. doi: 10.3390/fermentation11070389

[153] Inthamat P Siripatrawan U. Influence of chitosan encapsulation on functionality and stability of astaxanthin nanoemulsion fabricated using high pressure homogenization. Int J Biol Macromol. (2025) 303:140379. doi: 10.1016/j.ijbiomac.2025.140379, 39880245

[154] Lapčík L Lapčíková B Valenta T Vašina M Dudová P Fišera M. Study of natural dyes’ liposomal encapsulation in food dispersion model systems via high-pressure homogenization. Molecules. (2025) 30:1845. doi: 10.3390/molecules30081845, 40333878 PMC12029904

[155] Zhang X Lin X Wang J Luo X Xu B. Encapsulation of pterostilbene in hydroxypropyl-β-cyclodextrin using high-pressure homogenization and pH-shift methods: characterization, stability, and static in vitro digestion. J Food Eng. (2025) 401:112656. doi: 10.1016/j.jfoodeng.2025.112656

[156] Sareminia S Najafi A. Enhancing extrusion-Spheronization pharmaceutical production efficiency: an AI-driven approach to pellet formulation and manufacturing. J Pharm Innov. (2025) 20:113. doi: 10.1007/s12247-025-10028-1

[157] Taghizadeh Z Rakhshani S Jahani V Rajabi O Haghighi HM Abbaspour M. Preparation and in vitro characterization of carvacrol pellets by combination of liquisolid technique and extrusion-spheronization. J Drug Delivery Sci Technol. (2021) 61:102232. doi: 10.1016/j.jddst.2020.102232

[158] Jacobsen NMY Caglayan I Caglayan A Bar-Shalom D Müllertz A. Achieving delayed release of freeze-dried probiotic strains by extrusion, spheronization and fluid bed coating-evaluated using a three-step in vitro model. Int J Pharm. (2020) 591:120022. doi: 10.1016/j.ijpharm.2020.120022, 33122110

[159] Gangurde AB Sav AK Javeer SD Moravkar KK Pawar JN Amin PD. Modified extrusion-spheronization as a technique of microencapsulation for stabilization of choline bitartrate using hydrogenated soya bean oil. Int J Pharm Investig. (2015) 5:275–83. doi: 10.4103/2230-973X.167696, 26682198 PMC4675009

[160] Maboos M Yousuf RI Shoaib MH Nasiri I Hussain T Ahmed HF . Effect of lipid and cellulose based matrix former on the release of highly soluble drug from extruded/spheronized, sintered and compacted pellets. Lipids Health Dis. (2018) 17:136. doi: 10.1186/s12944-018-0783-8, 29885655 PMC5994249

[161] El-Saadony MT Saad AM Mohammed DM Alkafaas SS Abd El-Mageed TA Fahmy MA . Plant bioactive compounds: extraction, biological activities, immunological, nutritional aspects, food application, and human health benefits—a comprehensive review. Front Nutr. (2025) 12:1659743. doi: 10.3389/fnut.2025.1659743, 41487672 PMC12757306

[162] Multisona RR Jarzębski M Szwajca A Gramza-Michałowska A. Advancing microencapsulation strategies for bioactive compounds: enhancing stability, bioavailability, and controlled release in food applications. Nanotechnol Rev. (2025) 14:20250222. doi: 10.1515/ntrev-2025-0222

[163] Saini RK Ranjit A Sharma K Prasad P Shang X Gowda KGM . Bioactive compounds of citrus fruits: a review of composition and health benefits of carotenoids, flavonoids, limonoids, and terpenes. Antioxidants. (2022) 11:239. doi: 10.3390/antiox11020239, 35204122 PMC8868476

[164] Saini RK Khan MI Kumar V Shang X Lee JH Ko EY. Bioactive compounds of agro-industrial by-products: current trends, recovery, and possible utilization. Antioxidants. (2025) 14:650. doi: 10.3390/antiox14060650, 40563284 PMC12189145

[165] Kaseke T Chew SC Magangana TP Fawole OA. Elucidating the microencapsulation of bioactives from pomegranate fruit waste for enhanced stability, controlled release, biological activity, and application. Food Bioprocess Technol. (2025) 18:4222–50. doi: 10.1007/s11947-024-03689-2

[166] Ma L Li Z Li L Bi X Xiao G Li L. Edible beeswax-flaxseed oil nanostructured lipid carriers for improved β-sitosterol bioaccessibility: structural advantages and controlled release in dynamic digestion. Food Chem. (2025) 492:145492. doi: 10.1016/j.foodchem.2025.145492 40651141

[167] Umar M Zafar S Fikry M Medhe SV Rungraeng N. Non-covalent complexes of plant-based proteins-polysaccharides and their applications to stabilize the delivery systems for bioactive compounds. Food Rev Intl. (2026) 42:957–87. doi: 10.1080/87559129.2025.2509865

[168] Tripathy S Srivastav PP. Maize starch and β-cyclodextrin nanocarriers for encapsulation of *Centella asiatica* polyphenols: synthesis, physicochemical properties, and pH-responsive delivery. Food Chem. (2026) 507:148202. doi: 10.1016/j.foodchem.2026.148202 41650766

[169] Rostamabadi MM Falsafi SR Shafieiuon M Abbasi Z Falahati M Mohebi Z . A robust emulsion gel based on κ-carrageenan and dialdehyde starch for stimuli-responsive of hydrophobic bioactive agents. Int J Biol Macromol. (2025) 318:145328. doi: 10.1016/j.ijbiomac.2025.145328, 40532990

[170] Abtahi NA Naghib SM Ghalekohneh SJ Mohammadpour Z Nazari H Mosavi SM . Multifunctional stimuli-responsive niosomal nanoparticles for co-delivery and co-administration of gene and bioactive compound: in vitro and in vivo studies. Chem Eng J. (2022) 429:132090. doi: 10.1016/j.cej.2021.132090

[171] Iannaco MC Mottola S De Marco I. Emulsification-driven size control in supercritical PLGA-based encapsulation: from nanocapsules to microcapsules. Powder Technol. (2025) 470:122016. doi: 10.1016/j.powtec.2025.122016

[172] Nunekpeku X Li H Zahid A Li C Zhang W. Advances in hydrogel-integrated SERS platforms: innovations, applications, challenges, and future prospects in food safety detection. Biosensors. (2025) 15:363. doi: 10.3390/bios15060363, 40558445 PMC12190936

[173] Kim SJ Kim DY Jeong D Lee C Cho HD Kim MP. Food-grade microgels for age-related macular degeneration: design, fabrication, and targeted delivery. Gels. (2026) 12:252. doi: 10.3390/gels12030252, 41892574 PMC13025106

[174] Rana H Gohel S Rabari P Patel D Thakkar V Gandhi T. Design and development of prolong-acting intelligent Nano-depot (PaIND) for improving patient adherence. AAPS PharmSciTech. (2026) 27:161. doi: 10.1208/s12249-026-03397-4, 41882271

[175] Yu M Chi C Chen L Li X. Tailoring smart Oral Nano/micro delivery system to address multifaceted gastrointestinal barriers for improved delivery of food bioactive compounds. Compr Rev Food Sci Food Saf. (2025) 24:e70275. doi: 10.1111/1541-4337.70275, 40976936

[176] Yammine J Chihib NE Gharsallaoui A Ismail A Karam L. Advances in essential oils encapsulation: development, characterization and release mechanisms. Polym Bull. (2024) 81:3837–82. doi: 10.1007/s00289-023-04916-0

[177] Gruber D Ruiz-Agudo C Rao A Pasler S Cölfen H Sturm EV. Complex coacervates: from polyelectrolyte solutions to multifunctional hydrogels for bioinspired crystallization. Crystals. (2024) 14:959. doi: 10.3390/cryst14110959

[178] Mankan E Karakas CY Saroglu O Mzoughi M Sagdic O Karadag A. Food-grade liposome-loaded delivery systems: current trends and future perspectives. Foods. (2025) 14:2978. doi: 10.3390/foods14172978, 40941093 PMC12428440

[179] Cao Y Dong X Chen X. Polymer-modified liposomes for drug delivery: from fundamentals to applications. Pharmaceutics. (2022) 14:778. doi: 10.3390/pharmaceutics14040778, 35456613 PMC9026371

[180] Yong SXM Song CP Choo WS. Impact of high-pressure homogenization on the extractability and stability of phytochemicals. Front Sustain Food Syst. (2021) 4:593259. doi: 10.3389/fsufs.2020.593259

[181] Morán D Gutiérrez G Blanco-López MC Marefati A Rayner M Matos M. Synthesis of starch nanoparticles and their applications for bioactive compound encapsulation. Appl Sci. (2021) 11:4547. doi: 10.3390/app11104547

[182] Marta H Rizki DI Mardawati E Djali M Mohammad M Cahyana Y. Starch nanoparticles: preparation, properties and applications. Polymers. (2023) 15:1167. doi: 10.3390/polym15051167, 36904409 PMC10007494

[183] Garg T Arora S Pahwa R. Cellulose and its derivatives: structure, modification, and application in controlled drug delivery. Fut J Pharm Sci. (2025) 11:76. doi: 10.1186/s43094-025-00834-2

[184] Huang X Li T Li S. Encapsulation of vitexin-rhamnoside based on zein/pectin nanoparticles improved its stability and bioavailability. Curr Res Food Sci. (2023) 6:100419. doi: 10.1016/j.crfs.2022.100419, 36582445 PMC9792296

[185] Chicea D Nicolae-Maranciuc A. A review of chitosan-based materials for biomedical, food, and water treatment applications. Materials. (2024) 17:5770. doi: 10.3390/ma17235770, 39685206 PMC11642024

[186] Yarahmadi A Dousti B Karami-Khorramabadi M Afkhami H. Materials based on biodegradable polymers chitosan/gelatin: a review of potential applications. Front Bioeng Biotechnol. (2024) 12:1397668. doi: 10.3389/fbioe.2024.1397668, 39157438 PMC11327468

[187] Nezamdoost-Sani N Khaledabad MA Amiri S Khaneghah AM. Alginate and derivatives hydrogels in encapsulation of probiotic bacteria: an updated review. Food Biosci. (2023) 52:102433. doi: 10.1016/j.fbio.2023.102433

[188] Kadirvel V Narayana GP. Edible gums—an extensive review on its diverse applications in various food sectors. Food Bioeng. (2023) 2:384–405. doi: 10.1002/fbe2.12067

[189] Froelich A Jakubowska E Jadach B Gadziński P Osmałek T. Natural gums in drug-loaded micro-and nanogels. Pharmaceutics. (2023) 15:759. doi: 10.3390/pharmaceutics15030759, 36986620 PMC10059891

[190] Singh R Priya H Kumar SR Trivedi D Prasad N Ahmad F . Gum ghatti: a comprehensive review on production, processing, remarkable properties, and diverse applications. ACS Omega. (2024) 9:9974–90. doi: 10.1021/acsomega.3c08198, 38463282 PMC10918680

[191] Minj S Anand S. Whey proteins and its derivatives: bioactivity, functionality, and current applications. Dairy. (2020) 1:233–58. doi: 10.3390/dairy1030016

[192] Sadiq U Gill H Chandrapala J. Casein micelles as an emerging delivery system for bioactive food components. Foods. (2021) 10:1965. doi: 10.3390/foods10081965, 34441743 PMC8392355

[193] Zahariev N Draganova M Zagorchev P Pilicheva B. Casein-based nanoparticles: a potential tool for the delivery of daunorubicin in acute lymphocytic leukemia. Pharmaceutics. (2023) 15:471. doi: 10.3390/pharmaceutics15020471, 36839793 PMC9967267

[194] Raynes JK Mata J Wilde KL Carver JA Kelly SM Holt C. Structure of biomimetic casein micelles: critical tests of the hydrophobic colloid and multivalent-binding models using recombinant deuterated and phosphorylated β-casein. J. Struct Biol. (2024) 9:100096. doi: 10.1016/j.yjsbx.2024.100096 PMC10840362 38318529

[195] Joy JM Padmaprakashan A Pradeep A Paul PT Mannuthy RJ Mathew S. A review on fish skin-derived gelatin: elucidating the gelatin peptides—preparation, bioactivity, mechanistic insights, and strategies for stability improvement. Foods. (2024) 13:2793. doi: 10.3390/foods13172793, 39272559 PMC11394984

[196] Song L Chen Y Liu H Zhang X. Preparation, biological activities, and potential applications of hen egg-derived peptides: a review. Foods. (2024) 13:885. doi: 10.3390/foods13060885, 38540877 PMC10969619

[197] Zhao P Ji Y Yang H Meng X Liu B. Soy protein isolate–chitosan nanoparticle-stabilized Pickering emulsions: stability and in vitro digestion for DHA. Mar Drugs. (2023) 21:546. doi: 10.3390/md21100546, 37888481 PMC10608249

[198] Gong H Fu H Zhang J Zhang Q Wang Y Wang D . Preparation of soybean protein-based nanoparticles and its application as encapsulation carriers of bioactive substances. Lwt. (2024) 191:115680. doi: 10.1016/j.lwt.2023.115680

[199] Harasym J Banaś K. Lecithin’s roles in oleogelation. Gels. (2024) 10:169. doi: 10.3390/gels10030169 38534587 PMC10969852

[200] Rahim MA Zahran HA Jaffar HM Ambreen S Ramadan MF Al-Asmari F . Liposomal encapsulation in food systems: a review of formulation, processing, and applications. Food Sci Nutr. (2025) 13:e70587. doi: 10.1002/fsn3.70587, 40766785 PMC12321603

[201] Babarabie M Sardoei AS Jamali B Hatami M. Carnauba wax-based edible coatings retain quality enhancement of orange (*Citrus sinensis* cv. Moro) fruits during storage. Sci Rep. (2024) 14:4133. doi: 10.1038/s41598-024-54556-1, 38374381 PMC10876575

[202] Younes M Aggett P Aguilar F Crebelli R Dusemund B Lambré C. Re-evaluation of mono-and di-glycerides of fatty acids (E 471) as food additives. EFSA J. (2017) 15:e05045. doi: 10.2903/j.efsa.2017.5045, 32625340 PMC7010209

[203] Zheng J Liang Y Li J Lin S Zhang Q Zuo K . Enzymatic preparation of mono-and diacylglycerols: a review. Grain Oil Sci Technol. (2023) 6:185–205. doi: 10.1016/j.gaost.2023.10.002

[204] Şahansoy H Caner C Yüceer M. The shellac and shellac nanocomposite coatings on enhanced the storage stability of fresh eggs for sustainable packaging. Int J Biol Macromol. (2024) 261:129817. doi: 10.1016/j.ijbiomac.2024.129817, 38286370

[205] Brudzyńska P Sionkowska A. The effects of shellac and glycerol on the physicochemical properties of chitosan films. Polymers. (2025) 17:1298. doi: 10.3390/polym17101298, 40430594 PMC12114691

[206] de Moura SCSR Alvim ID. "Ionotropic gelation". In: Bioactives Encapsulation: Food Applications. (eds). Cristina de Pinho S Favaro-Trindade CS. New York: Springer US (2025). p. 43–65.

[207] Eghbal N Choudhary R. Complex coacervation: encapsulation and controlled release of active agents in food systems. Lwt. (2018) 90:254–64. doi: 10.1016/j.lwt.2017.12.036

[208] Dehghani F Farhadian N. "Encapsulation: fluidized bed coating technology". In: Principles of Biomaterials Encapsulation: Volume One. (eds). Sefat F Farzi G Mozafari M. Woodhead Publishing (2023). p. 143–56.

[209] Vinchhi P Patel JK Patel MM. "High-pressure homogenization techniques for nanoparticles". In: Emerging Technologies for Nanoparticle Manufacturing. (eds). Patel JK Pathak YV. Cham: Springer International Publishing (2021). p. 263–85.

[210] Nguyen TTL Anton N Vandamme TF. Nutraceutical compounds encapsulated by extrusion–spheronization. New Polymers Encapsul Nutraceut Compounds. (2016) 16:195–230. doi: 10.1002/9781119227625.ch9

[211] Żurek N Świeca M Pawłowska A Kapusta IT. Microencapsulation of blueberry (*Vaccinium myrtillus* L.) extracts via ionotropic gelation: in vitro assessment of bioavailability of phenolic compounds and their activity against colon cancer cells. Appl Sci. (2024) 14:7842. doi: 10.3390/app14177842

[212] Mukherjee P Baruah KN Uppaluri RV. Encapsulation and characterization of commercial green tea extracts using green methods: a comparative study of inclusion complexation and ion gelation. J Food Meas Charact. (2024) 18:916–29. doi: 10.1007/s11694-023-02250-7

[213] Gallegos-Tintoré S May-Canché M Chel-Guerrero L Castellanos-Ruelas A Betancur-Ancona D. Preservation by ionic gelation encapsulation of the antioxidant activity of protein hydrolysate derived from lionfish (*Pterois volitans*, L.) muscle proteins. Food Sci Biotechnol. (2024) 33:2979–87. doi: 10.1007/s10068-024-01557-5, 39220316 PMC11364726

[214] Farrell M dos Santos Lima A Mohammadi N Cruz TM Zhou F Xu YQ . Microencapsulation of purple tea polyphenols using the vibrating nozzle ionotropic gelation technology: metabolomics, bioactivity, and application in milky tea. LWT. (2024) 199:116099. doi: 10.1016/j.lwt.2024.116099

[215] Silveira MP Almeida FLC Alvim ID Prata AS. Encapsulation of pomegranate polyphenols by ionic gelation: strategies for improved retention and controlled release. Food Res Int. (2023) 174:113590. doi: 10.1016/j.foodres.2023.113590, 37986529

[216] Celebioglu A Topuz F Aboelkheir M Uyar T. Electrospun Gelatin Nanofibers Encapsulating Cyclodextrin–Eugenol and Cyclodextrin–Thymol Inclusion Complexes for Nutraceutical Delivery. Washington, DC: ACS Food Science & Technology (2026).

[217] González-Cruz EM Calderón-Santoyo M Chevalier-Lucia D Picart-Palmade L Calderón-Chiu C Andrade-González I . Use of the acid fraction of potato protein to encapsulate bioactive compounds through Nanofibers obtained by electrospinning process. ACS Food Sci Technol. (2025) 5:812–21. doi: 10.1021/acsfoodscitech.4c01000

[218] Ma J Tan Z Wu M Tian Z Xu C Zhang J . Co-encapsulation of probiotic Lactiplantibacillus plantarum and polyphenol within novel polyvinyl alcohol/fucoidan electrospun nanofibers with improved viability and antioxidation. Int J Biol Macromol. (2024) 282:136907. doi: 10.1016/j.ijbiomac.2024.136907, 39476917

[219] Falsafi SR Rostamabadi H Nishinari K Amani R Jafari SM. The role of emulsification strategy on the electrospinning of β-carotene-loaded emulsions stabilized by gum Arabic and whey protein isolate. Food Chem. (2022) 374:131826. doi: 10.1016/j.foodchem.2021.131826, 34915375

[220] Ghasemi M Miri MA Najafi MA Tavakoli M Hadadi T. Encapsulation of cumin essential oil in zein electrospun fibers: characterization and antibacterial effect. J Food Meas Charact. (2022) 16:1613–24. doi: 10.1007/s11694-021-01268-z

[221] Rezazadeh A Bazardeh ME Ghasempour Z Kia EM. Gelatin/pectin complex coacervation for encapsulation of microwave-assisted extraction of bioactive compounds from red onion skin. Int J Biol Macromol. (2025) 319:145416. doi: 10.1016/j.ijbiomac.2025.145416, 40545074

[222] Ayar-Sumer EN Nyambe C Hashim MA Altin-Yavuzarslan G El-Messery TM Ozçelik B. Optimizing encapsulation of black carrot extract using complex coacervation technique: maximizing the bioaccessibility and release kinetics in different food matrixes. Lwt. (2024) 198:115995. doi: 10.1016/j.lwt.2024.115995

[223] Devi LM Das AB Badwaik LS. Ultrasound-assisted extraction of anthocyanin from black rice bran and its encapsulation by complex coacervation. Food Hydrocoll Health. (2024) 5:100174. doi: 10.1016/j.fhfh.2023.100174

[224] Jamshidian H Rafe A. Complex coacervate of wheat germ protein/high methoxy pectin in encapsulation of d-limonene. Chem Biol Technol Agric. (2024) 11:60. doi: 10.1186/s40538-024-00579-9

[225] Mu J Hu R Tang Y Dong W Zhang Z. Microencapsulation of green coffee oil by complex coacervation of soy protein isolate, sodium casinate and polysaccharides: physicochemical properties, structural characterisation, and oxidation stability. Int J Biol Macromol. (2024) 256:128064. doi: 10.1016/j.ijbiomac.2023.128064, 37967606

[226] Sukri N Putri TTM Mahani N Nurhadi B. Characteristics of propolis encapsulated with gelatin and sodium alginate by complex coacervation method. Int J Food Prop. (2023) 26:696–707. doi: 10.1080/10942912.2023.2179635

[227] Farnad N Farhadi K. Simple and complex coacervation methods for the nanoencapsulation of Rosa damascena mill L. anthocyanin in zein/potato starch: a new approach to enhance antioxidant and thermal properties. J Food Sci. (2023) 88:1019–32. doi: 10.1111/1750-3841.16463, 36658670

[228] Preradović N Nakarada Đ Gašić U Simonović Radosavljević J Mojović M. Green extraction and liposomal encapsulation of Inonotus obliquus (Chaga) extracts: comparative phytochemical and antioxidant analysis. Molecules. (2026) 31:146. doi: 10.3390/molecules31010146, 41515441 PMC12788109

[229] Prevete G Carvalho LG del Carmen Razola-Diaz M Verardo V Mancini G Fiore A . Ultrasound assisted extraction and liposome encapsulation of olive leaves and orange peels: how to transform biomass waste into valuable resources with antimicrobial activity. Ultrason Sonochem. (2024) 102:106765. doi: 10.1016/j.ultsonch.2024.106765, 38232412 PMC10827538

[230] Prevete G Donati E Ruggiero AP Fardellotti S Lilla L Ramundi V . Encapsulation of *olea europaea* leaf polyphenols in liposomes: a study on their antimicrobial activity to turn a byproduct into a tool to treat bacterial infection. ACS Appl Mater Interfaces. (2024) 16:68850–63. doi: 10.1021/acsami.4c13302, 39631768 PMC11660030

[231] Ahmed SA Saleem MF Hassanzadeh H. Optimization of solvent evaporation method in liposomal nanocarriers loaded-garlic essential oil (*Allium sativum*): based on the encapsulation efficiency, antioxidant capacity, and instability. IET Nanobiotechnol. (2023) 17:438–49. doi: 10.1049/nbt2.12142, 37277887 PMC10374552

[232] Yan Y Song D He L Zhao Y Wang L Wang X . Optimizing stability and controlled release of co-encapsulated probiotics and vitamin B12 using fluidized bed coating: impact of plasticizers on tailored HPMCAS-coated granules. Curr Res Food Sci. (2025) 11:101191. doi: 10.1016/j.crfs.2025.101191 40978511 PMC12446386

[233] Budin AC Wensing CS Cruz CLV Ruffi CRG Garcia AO de Moura SCSR. Stability study of bioactive compounds from yerba mate extract encapsulated by ionic gelation and application of microparticles in fruit and cereal bars. LWT. (2025) 215:117245. doi: 10.1016/j.lwt.2024.117245

[234] Morales-Huerta A Flores-Andrade E Jiménez-Fernández M Beristain CI Pascual-Pineda LA. Microencapsulation of betalains by foam fluidized drying. J Food Eng. (2023) 359:111701. doi: 10.1016/j.jfoodeng.2023.111701

[235] Afshari K Javanmard Dakheli M Ramezan Y Bassiri A Ahmadi Chenarbon H. Physicochemical and control releasing properties of date pit (*Phoenix dactylifera* L.) phenolic compounds microencapsulated through fluidized-bed method. Food Sci Nutr. (2023) 11:1367–82. doi: 10.1002/fsn3.3173, 36911813 PMC10003029

[236] Semenoglou I Katsouli M Giannakourou M Taoukis P. Effect of high-pressure homogenization and wall material composition on the encapsulation of polyunsaturated fatty acids from fish processing. Molecules. (2025) 30:1434. doi: 10.3390/molecules30071434, 40286064 PMC11990714

[237] Chen F Lu M Rogers MA Cao Y Lan Y. Curcumin loading in camellia seed oil body and multi-compartment emulsion fabrication: effect of high-pressure homogenization pretreatment. Food Res Int. (2025) 225:118091. doi: 10.1016/j.foodres.2025.118091 41508504

[238] Tuo Y Wang M Yu Y Li Y Hu X Wu L . Enhancing Fucoxanthin Pickering emulsion stability and encapsulation with seaweed cellulose Nanofibrils using high-pressure homogenization. Mar Drugs. (2025) 23:311. doi: 10.3390/md23080311, 40863629 PMC12387411

[239] Gu L Ji S Wu B Wang W Cheng J Xia Q. Astaxanthin encapsulation in nanocapsule by high-pressure homogenization technology: a study on stability, antioxidant activity and in vitro release. J Dispers Sci Technol. (2024) 45:1129–40. doi: 10.1080/01932691.2023.2199830

[240] Wong HT Pan P Wu Z Sarojini V. Development of biodegradable controlled-release anti-Phytophthora formulations using poly (3-hydroxybutyrate-co-3-hydroxyvalerate). ACS Agric Sci Technol. (2026) 6:575–85. doi: 10.1021/acsagscitech.5c00834

[241] Aghrbi I Fülöp V Jakab G Kallai-Szabo N Balogh E Antal I. Nanosuspension with improved saturated solubility and dissolution rate of cilostazol and effect of solidification on stability. J Drug Delivery Sci Technol. (2021) 61:102165. doi: 10.1016/j.jddst.2020.102165

[242] Hung SF Hsieh CM Chen YC Lin CM Ho HO Sheu MT. Formulation and process optimization of multiparticulate pulsatile system delivered by osmotic pressure-activated rupturable membrane. Int J Pharm. (2015) 480:15–26. doi: 10.1016/j.ijpharm.2015.01.006, 25575473

[243] Yadav D Survase S Kumar N. Dual coating of swellable and rupturable polymers on Glipizide loaded MCC pellets for pulsatile delivery: formulation design and in vitro evaluation. Int J Pharm. (2011) 419:121–30. doi: 10.1016/j.ijpharm.2011.07.026, 21807081

[244] Zhang Y Guo Y Liu F Luo Y. Recent development of egg protein fractions and individual proteins as encapsulant materials for delivery of bioactives. Food Chemistry. (2023) 403:134353. 36179637 10.1016/j.foodchem.2022.134353

